# Spatial and Seasonal Distribution of American Whaling and Whales in the Age of Sail

**DOI:** 10.1371/journal.pone.0034905

**Published:** 2012-04-27

**Authors:** Tim D. Smith, Randall R. Reeves, Elizabeth A. Josephson, Judith N. Lund

**Affiliations:** 1 World Whaling History, Redding, California, United States of America; 2 Okapi Wildlife Associates, Hudson, Quebec, Canada; 3 Integrated Statistics, Woods Hole, Massachusetts, United States of America; 4 New Bedford Whaling Museum, New Bedford, Massachusetts, United States of America; University of Alberta, Canada

## Abstract

American whalemen sailed out of ports on the east coast of the United States and in California from the 18^th^ to early 20^th^ centuries, searching for whales throughout the world’s oceans. From an initial focus on sperm whales (*Physeter macrocephalus*) and right whales (*Eubalaena* spp.), the array of targeted whales expanded to include bowhead whales (*Balaena mysticetus*), humpback whales (*Megaptera novaeangliae*), and gray whales (*Eschrichtius robustus*). Extensive records of American whaling in the form of daily entries in whaling voyage logbooks contain a great deal of information about where and when the whalemen found whales. We plotted daily locations where the several species of whales were observed, both those caught and those sighted but not caught, on world maps to illustrate the spatial and temporal distribution of both American whaling activity and the whales. The patterns shown on the maps provide the basis for various inferences concerning the historical distribution of the target whales prior to and during this episode of global whaling.

## Introduction

The world has changed a great deal over the last few centuries, and this truism extends to the numbers, diversity, and spatial occurrence of creatures in the sea [1]–[3]. Although it is difficult to decide what to regard as a “natural” or baseline state of the world’s fauna and flora, it is reasonable to hypothesize that because of intense whaling by many nations over the past several centuries [4], we are a long way from seeing fully “recovered” whale populations, either numerically or spatially.

There have been several attempts to measure the initial and residual effects of whaling numerically, usually by estimating how many whales were likely removed by whaling and combining that information with information on the dynamics of whale populations and on how large the living populations are today e.g., [5], . There have also been some attempts to describe the spatial effects of whaling regionally [7]–[9] and globally [10]–[13]. While numerical effects are of course relevant [14], the spatial effects on regional populations are also important in determining present status.

In the 19^th^ century the American whaling industry was in its most malignant phase, spreading literally to the ends of the earth in search of its quarry. One whale population after another was depleted, often with remarkable rapidity. For example, right whales in the North Pacific and around New Zealand were greatly reduced within a decade [15]–[16]. While American vessels dominated offshore whaling in the 19^th^ century, substantial numbers of British and French whaling ships were active as were a number of shore-based whaling stations worldwide [4].

American whalemen focused on seven species of whales in five genera: the sperm whale (*Physeter macrocephalus*), the bowhead whale (*Balaena mysticetus*), the humpback whale (*Megaptera novaeangliae*), the gray whale (*Eschrichtius robustus*), the southern right whale (*Eubalaena australis*), the North Atlantic right whale (*Eubalaena glacialis*), and the North Pacific right whale (*Eubalaena japonica*). The whalemen distinguished the first four species, using a variety of recognizable names. The whalemen did not distinguish among the three right whale species in their logbooks, referring to all members of the genus *Eubalaena* simply as right whales, but the species involved can be inferred geographically as they are spatially disjunct.

Daily logbooks and journals (both termed logbooks here for simplicity) kept by American whalemen document the carnage. These records of whaling voyages were useful to whalemen, ship owners, and agents as evidence of the most promising areas for whaling. Logbooks from many American whaling voyages have been preserved [17] in public and private collections. The first large-scale collection of data from logbooks for scientific purposes was led by LCDR Matthew Fontaine Maury of the US Navy in Washington, D.C. during the 1840s [10].

Following Maury’s lead, in the 1920s Charles Haskins Townsend and his assistant Arthur C. Watson of the New York Zoological Society in New York also collected data from whaling logbooks [11]. Both Maury and Townsend used their data to illustrate the distribution of whales on global maps. We located and digitized the original data sheets of the Maury and Townsend studies.

**Figure 1:**
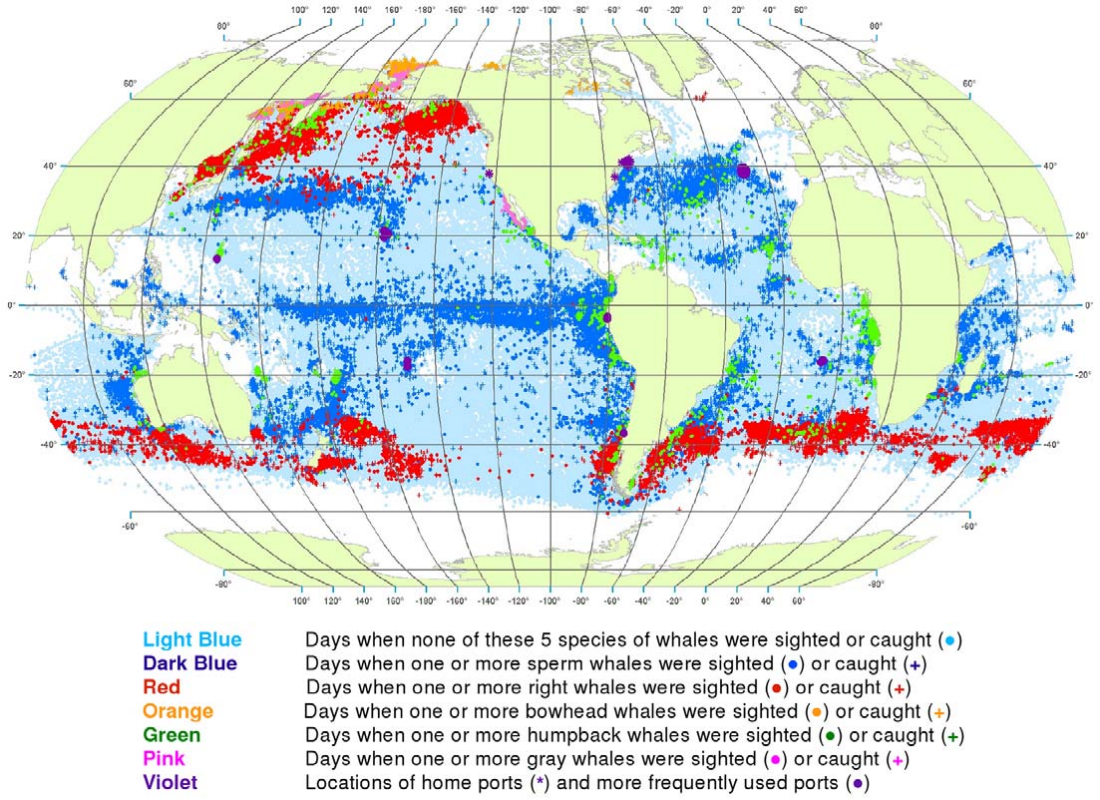
All observations of sperm, right, bowhead, gray, and humpback whales. Daily locations of vessels were extracted from a sample of American whaling logbooks for voyages departing between 1780 and 1920. Days with no whale observations and days with observations of sperm, right, bowhead, humpback, and gray whales and locations of key ports were distinguished by the colors indicated. Whalemen from other countries caught whales in many of the same areas and in some areas where American whalemen did not go (see text).

We also extracted data from American logbooks as part of a project sponsored by the Census of Marine Life (www.coml.org). The combined Maury, Townsend, and Census of Marine Life (CoML) data represent roughly 10% of the American whaling voyages between 1780 and 1920, when the vast majority of such voyages occurred. Details of the Maury and Townsend maps, the three data sources, and our treatment of the data are given under Materials and Methods, below.

We used these data to generate color-coded global maps of the daily locations of whaling vessels. Days with no whale observations and days with observations of sperm, right, bowhead, humpback, and gray whales were distinguished by different colors. These maps extend what was shown by Maury and Townsend, and better represent the spatial distribution of American whaling and the targeted whale populations.

## Results

### Geographic Distribution

From 1780 to 1920 American whalemen sought their prey in most of the world’s oceans, missing only a few areas as indicated by the white ocean regions in Figure 1. Although they whaled northward to the Arctic Ocean and along the ice edge to roughly 70°N latitude in the northern hemisphere, they did not venture nearly as far poleward in the southern hemisphere. American whalemen in the Atlantic Ocean searched mainly south of 50°N and north of 40°S, reaching farther southward along the South American coast and farther northward along the North American coast toward Hudson Bay. They also hunted whales across the Indian Ocean between 20° and 45°S and north to almost 20°N in the west. In the Pacific Ocean, they searched almost the entire basin.

These whalemen rarely visited some areas that are clearly identifiable in Figure 1, i.e., the northeastern North Atlantic, the western Caribbean Sea, the central and eastern Indian Ocean north of 10°S, waters north of Australia including the Java, Timor, Arafura, and western Coral seas, and waters south of southeastern Asia including the Bay of Bengal and the South China and Philippine seas, and areas south of roughly 50° to 60°S, variably around the globe. Some of these areas were not visited by American vessels but were by vessels of other nations, for example the northeastern North Atlantic (encompassing the Barents and Greenland seas), where European and British ship-based whalemen hunted right whales and bowhead whales, mostly before the 19^th^ century. Areas of lee shores and dangerous coral reefs were avoided because of the risks of sailing in these locations. Although vessels reached as far south as 60°S at the southern tip of South America in moving between the Atlantic and the Pacific, little whaling was done south of 50°S in most areas because of notoriously bad weather.

**Figure 2:**
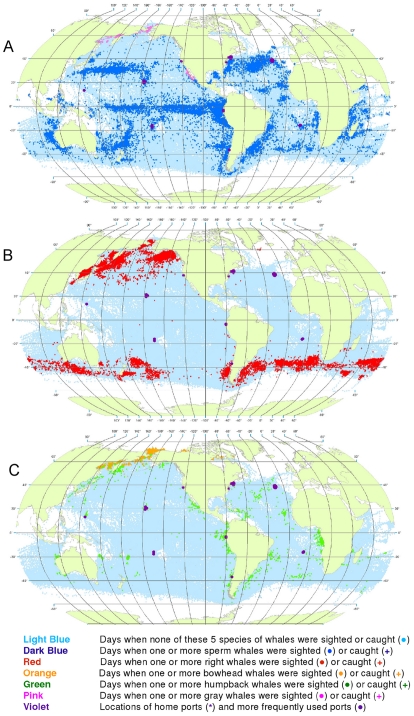
All observations of whales by groups of species. Daily locations of whaling vessels with observations of sperm and gray whales (A), right whales (B), and bowhead and humpback whales (C). The data were extracted from a sample of American whaling logbooks for voyages departing between 1780 and 1920. Days with no whale observations and days with observations of sperm, right, bowhead, humpback, and gray whales and locations of key ports were distinguished by the colors indicated.

American whalemen stopped at many ports during their voyages, visiting them to obtain provisions, replace crew, repair vessels, trans-ship whale products, and give crews opportunities to rest and relax. The ports most used during voyages in our data, including home ports, are shown for orientation in all maps. The use of individual ports varied considerably over time, depending on the changing geographic patterns of whaling.

Because American whalemen searched widely for whales, the locations where they observed the animals provide an indication of whale distribution at the time. The well-known patchiness of whale distribution is evident in Figure 1: the whales were found most consistently in particular regions, often referred to as whaling grounds [13]. An important example was the Japan Ground, stretching eastward from Japan along the 30°N latitude line (Figure 1). Of necessity, whalemen transited some areas but apparently found few or no whales: examples include a large portion of the offshore southeastern South Pacific south of 20°S, much of the central North Pacific between about 5°N and 25°N, and a sizable swath of the offshore Indian Ocean between roughly 20°S and 30°S.

The distributions of the targeted whale species overlap to a considerable degree, making it difficult to portray them all on a single map. Therefore we made separate maps for sperm and gray whales (Figure 2A), right whales alone (Figure 2B), and bowhead and humpback whales (Figure 2C). These provide a much clearer view of the general patterns for these species.

### Seasonal Distribution

Many species of whales exhibit seasonal changes in distribution associated with large-scale migratory movements. We therefore plotted the observations by season and, for more resolution, by month. These maps reveal considerable within-year variability in distribution patterns of both vessels and whales.

**Figure 3:**
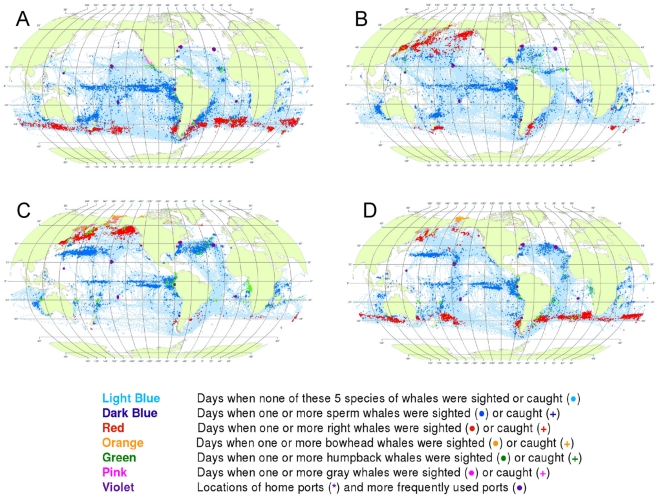
All observations of whales by season. Seasonal locations of whaling vessels in: December – February (A), March – May (B), June – August (C) and September – November (D). The data were extracted from a sample of American whaling logbooks for voyages departing between 1780 and 1920. Days with no whale observations and days with observations of sperm, right, bowhead, humpback, and gray whales and locations of key ports were distinguished by the colors indicated. The seasons were defined beginning with December to best reflect the similarities in distribution patterns within seasons.

Our whale maps by quarter of the year reveal some of the seasonal variability in the distribution of both whaleships and whales (Figures 3A–3D).

Sperm whales were present year-round in the Pacific, in bands along and slightly south of the equator, with little if any seasonal shift in latitude (Figures 3A–3D). Elsewhere the distribution of observations varied seasonally in complex ways. For example, in the southwestern Atlantic and southeastern Pacific sperm whales were observed along the coast of South America but primarily in the southern summer and fall (Figures 3A and 3B).

The distribution of southern right whales was nearly circumpolar in the southern spring and summer (September to February, Figures 3D and 3A). Although there was limited search effort during the southern fall and winter in the temperate latitudes of the southern hemisphere (Figures 3B and 3C), right whales were much less frequently seen. In all seasons there was a remarkable absence of right whales in the southeastern South Pacific over 45° of longitude. North Pacific right whales were present across much of the basin during at least the northern spring, summer, and fall (March to November, Figures 3B, 3C and 3D); there was essentially no search effort in temperate latitudes of the North Pacific during the winter (Figure 3A). There is no evidence in these maps that American whalemen located right whale calving grounds in the North Pacific. Our seasonal maps are not very informative for right whales in the North Atlantic because these whales had already been seriously depleted there by the late 18^th^ century, i.e., before large numbers of voyages represented by available logbooks occurred [18].

Bowhead whales were observed in the Sea of Okhotsk in the spring, summer, and fall (Figures 3B, 3C and 3D), in the Bering Sea in the spring and summer (Figures 3B and 3C), and north through the Bering Strait into the Chukchi and eastern Beaufort seas in the summer and fall (Figures 3C and 3D) [8]. American whalemen were less involved in the bowhead whale fishery in the eastern Arctic (centered in Davis Strait and Baffin Bay and around Svalbard) [19]–[21], therefore our maps are not informative on seasonal occurrence there. The records in the Hudson Bay region refer mainly to summer months (June to August, Figure 3C).

**Figure 4:**
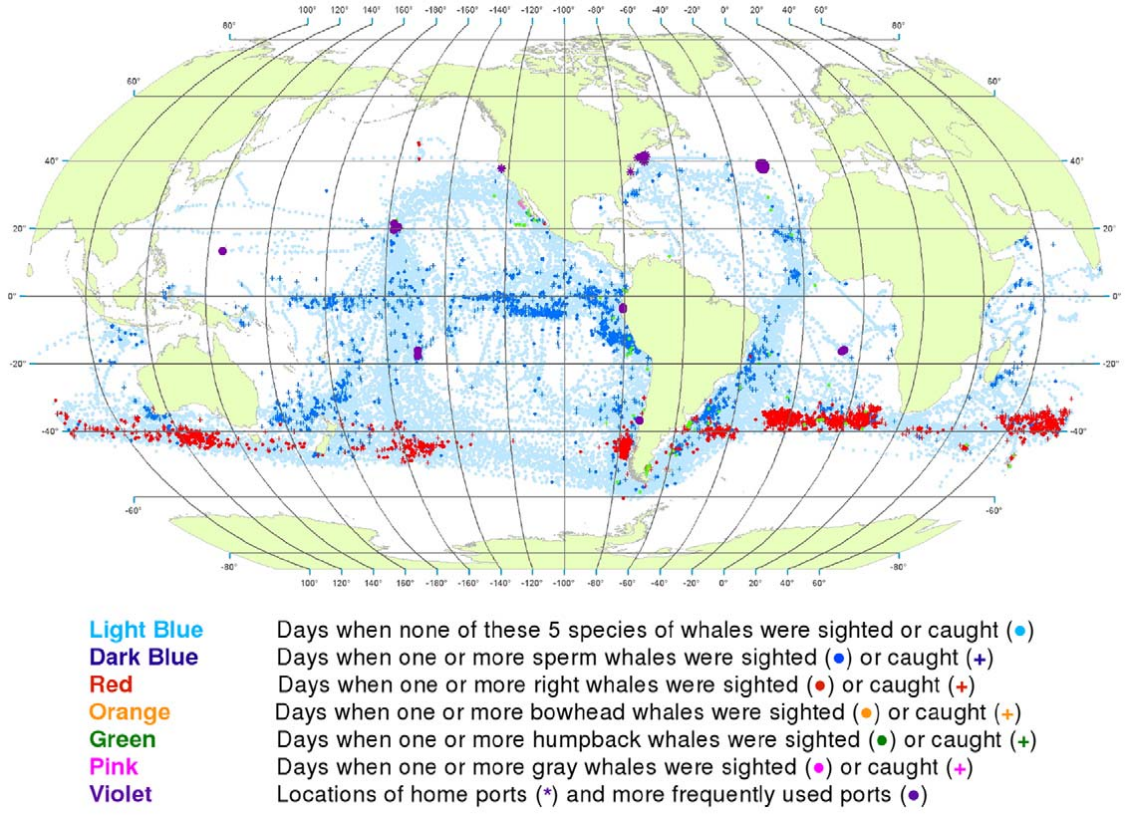
December observations of whales. The data were extracted from a sample of American whaling logbooks for voyages departing between 1780 and 1920. Days with no whale observations and days with observations of sperm, right, bowhead, humpback, and gray whales and locations of key ports were distinguished by the colors indicated.

**Figure 5:**
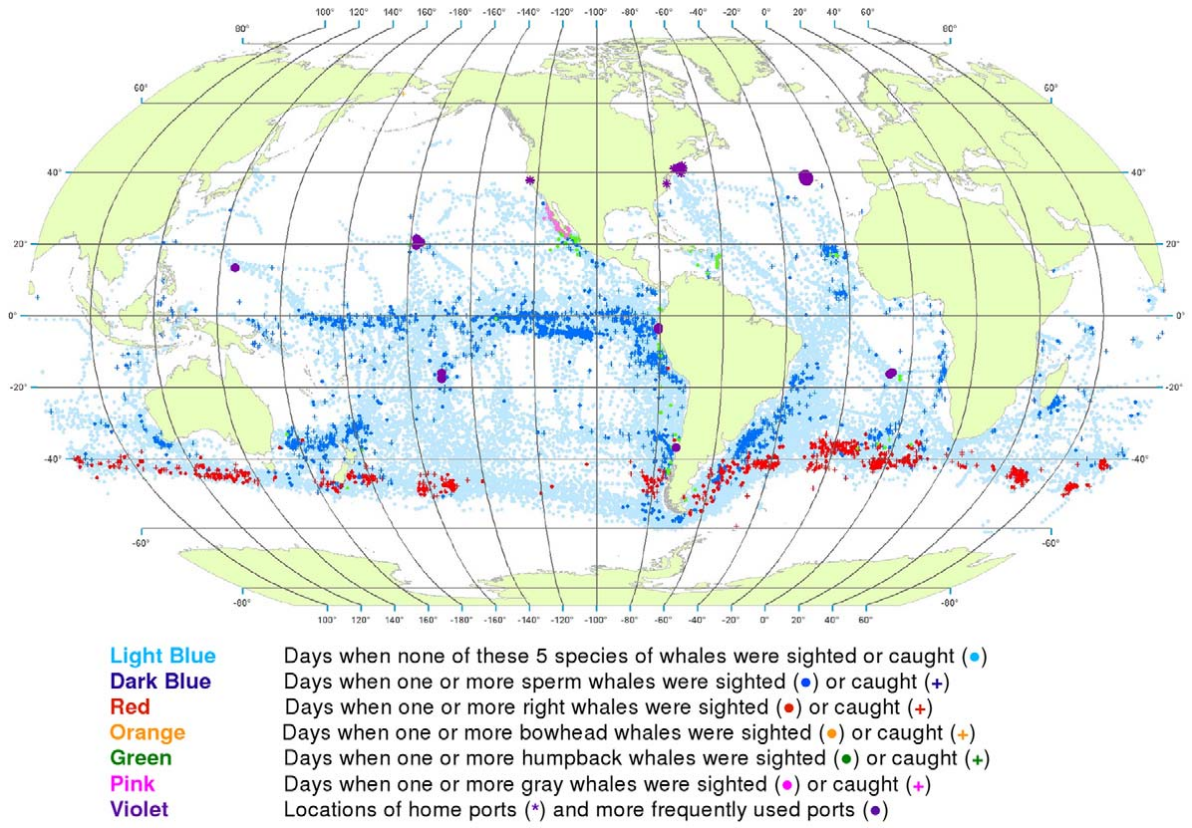
January observations of whales. The data were extracted from a sample of American whaling logbooks for voyages departing between 1780 and 1920. Days with no whale observations and days with observations of sperm, right, bowhead, humpback, and gray whales and locations of key ports were distinguished by the colors indicated.

The concentrations of humpback whale observations are broadly consistent with but not fully representative of what we know about the migrations of these animals between breeding and feeding areas. Humpbacks were observed mainly in tropical breeding and calving grounds in the winter and spring (Figure 3A and 3B for the northern hemisphere and Figures 3C and 3D for the southern hemisphere). In the northern hemisphere, they were seen in the spring and summer in feeding grounds north of 20°N, away from the breeding and calving grounds. In the North Pacific, humpback whales were observed as far as 60°N, while in the North Atlantic, American whalemen did not spend a lot of time north of 45°N and so did not often observe humpback whales in their more northerly feeding areas in that basin. Similarly, whalemen did not spend a lot of time south of 50°S where most humpback whales feed in the austral summer, apparently because of difficulty operating in those waters.

Gray whales were observed and hunted in winter breeding and calving areas along the west coast of Mexico (Figure 3A) and on their summer feeding grounds in the northern Bering Sea and Okhotsk Sea [22] (Figure 3C). They were not reported in the logbooks during their migrations to and from feeding areas. There is nothing in these data to suggest the American whalemen located the breeding and calving areas of gray whales on the western side of the North Pacific.

**Figure 6:**
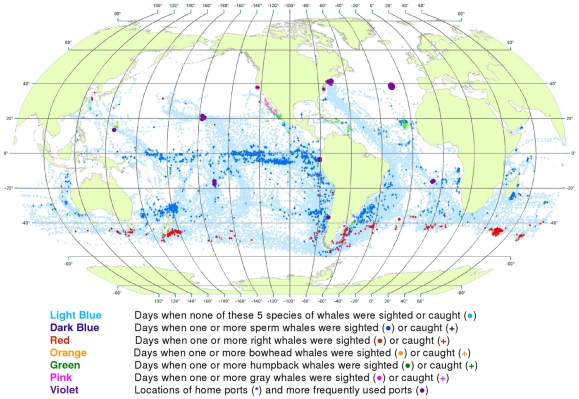
February observations of whales. The data were extracted from a sample of American whaling logbooks for voyages departing between 1780 and 1920. Days with no whale observations and days with observations of sperm, right, bowhead, humpback, and gray whales and locations of key ports were distinguished by the colors indicated.

**Figure 7:**
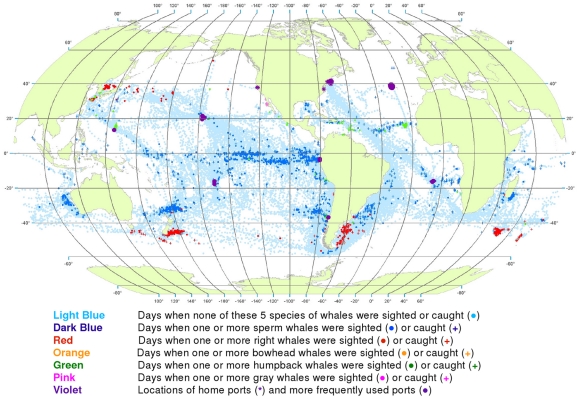
March observations of whales. The data were extracted from a sample of American whaling logbooks for voyages departing between 1780 and 1920. Days with no whale observations and days with observations of sperm, right, bowhead, humpback, and gray whales and locations of key ports were distinguished by the colors indicated.

### Monthly Distribution

The distribution of whales changed not just seasonally but often from month to month (Figures 4, 5, 6, 7, 8, 9, 10, 11, 12, 13, 14, and 15). For example, the nearly circumpolar distribution of southern right whales that is apparent in Figures 3A and 3D developed and subsided gradually from October to February (Figures 14, 15, 4, and 5).

**Figure 8:**
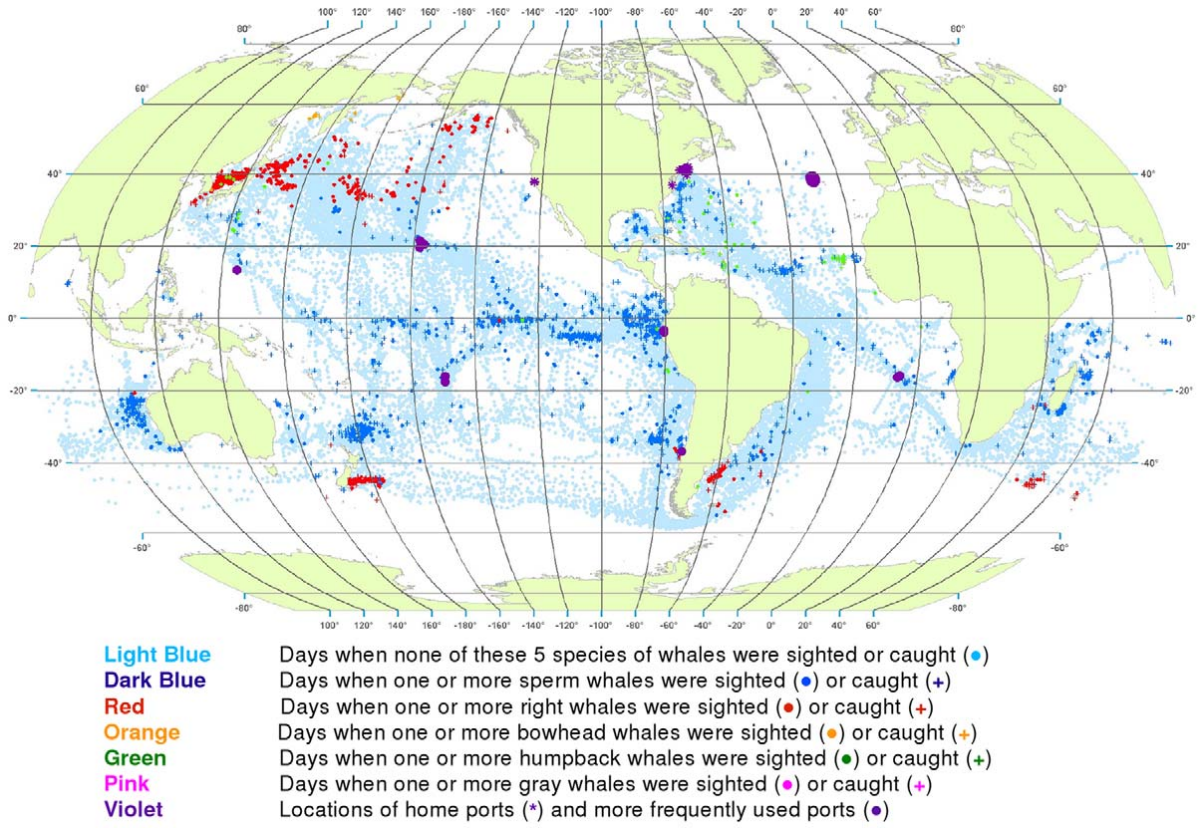
April observations of whales. The data were extracted from a sample of American whaling logbooks for voyages departing between 1780 and 1920. Days with no whale observations and days with observations of sperm, right, bowhead, humpback, and gray whales and locations of key ports were distinguished by the colors indicated.

**Figure 9:**
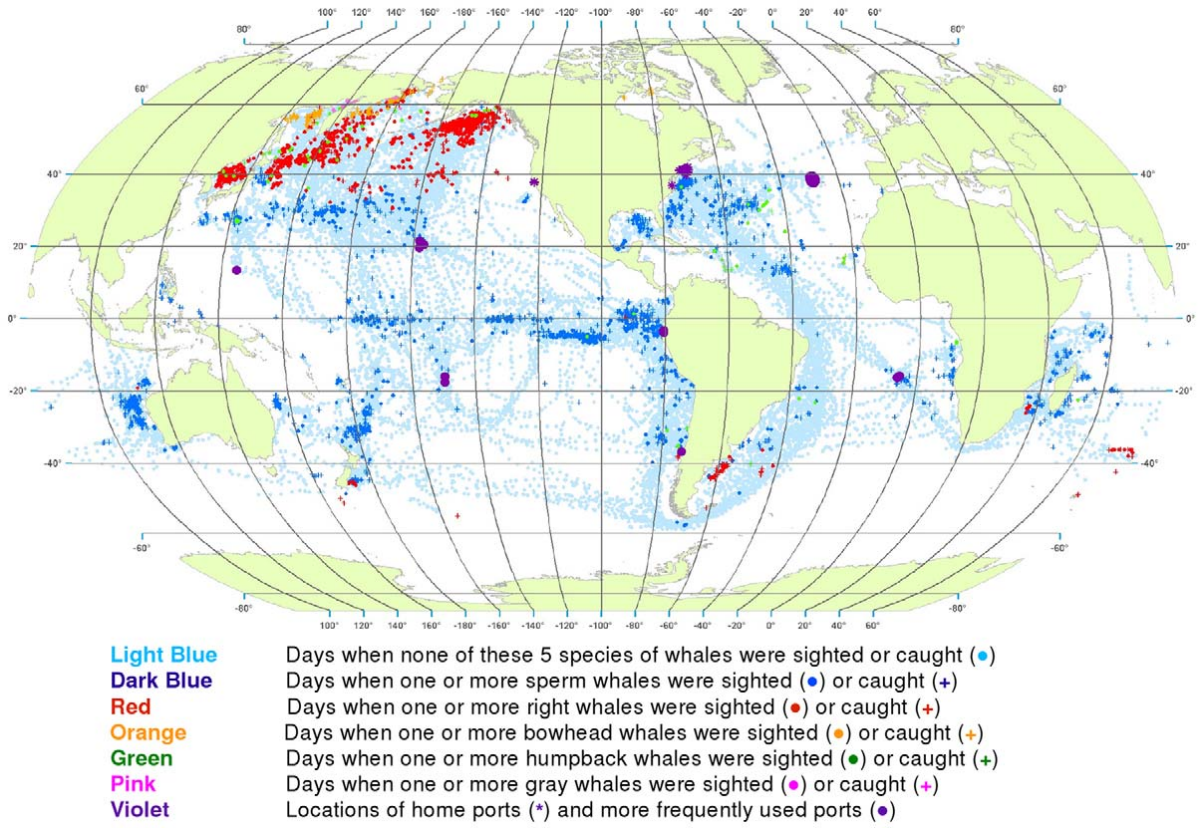
May observations of whales. The data were extracted from a sample of American whaling logbooks for voyages departing between 1780 and 1920. Days with no whale observations and days with observations of sperm, right, bowhead, humpback, and gray whales and locations of key ports were distinguished by the colors indicated.

**Figure 10:**
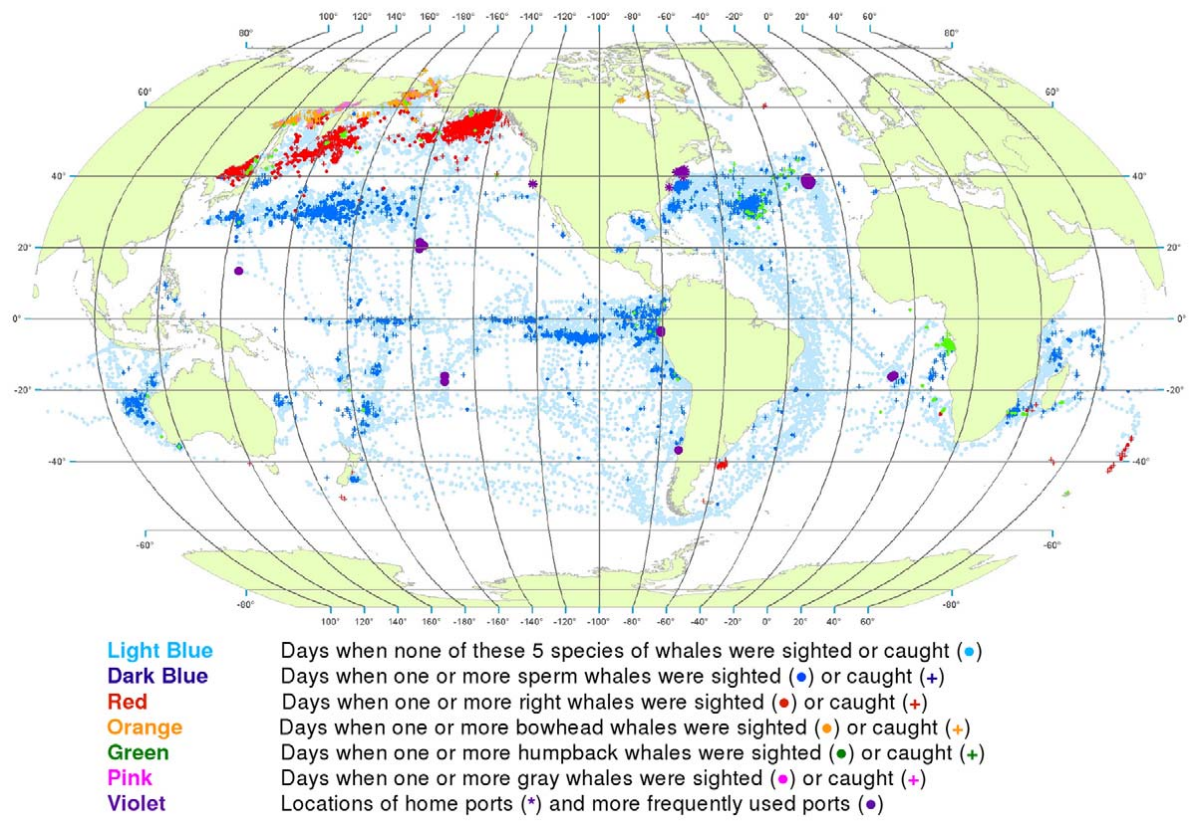
June observations of whales. The data were extracted from a sample of American whaling logbooks for voyages departing between 1780 and 1920. Days with no whale observations and days with observations of sperm, right, bowhead, humpback, and gray whales and locations of key ports were distinguished by the colors indicated.

**Figure 11:**
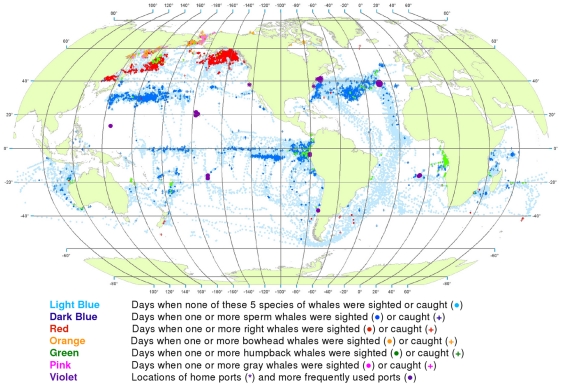
July observations of whales. The data were extracted from a sample of American whaling logbooks for voyages departing between 1780 and 1920. Days with no whale observations and days with observations of sperm, right, bowhead, humpback, and gray whales and locations of key ports were distinguished by the colors indicated.

**Figure 12:**
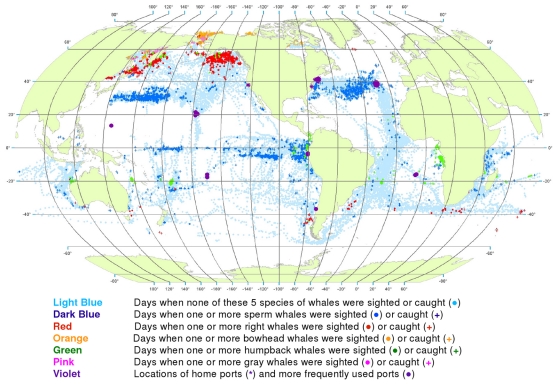
August observations of whales. The data were extracted from a sample of American whaling logbooks for voyages departing between 1780 and 1920. Days with no whale observations and days with observations of sperm, right, bowhead, humpback, and gray whales and locations of key ports were distinguished by the colors indicated.

**Figure 13:**
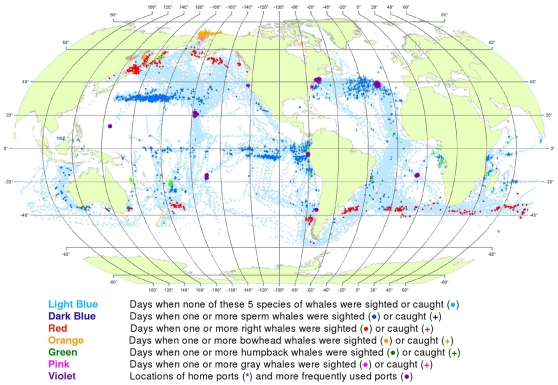
September observations of whales. The data were extracted from a sample of American whaling logbooks for voyages departing between 1780 and 1920. Days with no whale observations and days with observations of sperm, right, bowhead, humpback, and gray whales and locations of key ports were distinguished by the colors indicated.

**Figure 14:**
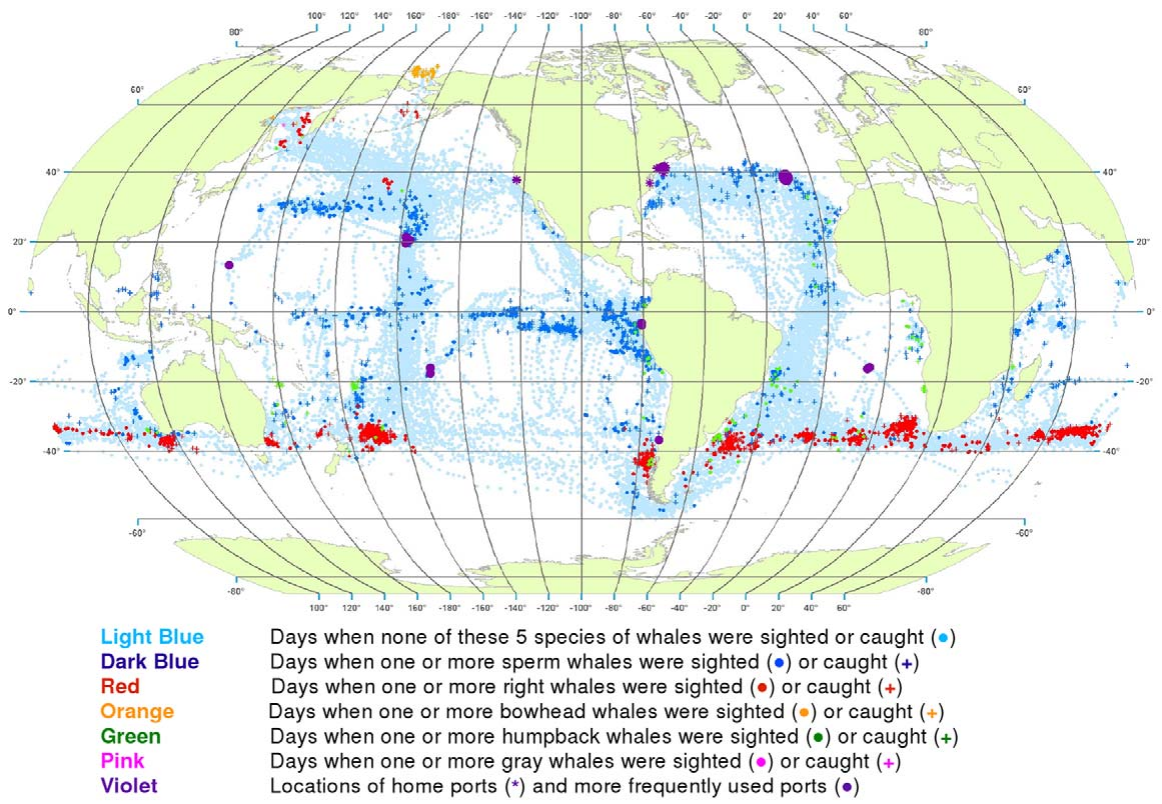
October observations of whales. The data were extracted from a sample of American whaling logbooks for voyages departing between 1780 and 1920. Days with no whale observations and days with observations of sperm, right, bowhead, humpback, and gray whales and locations of key ports were distinguished by the colors indicated.

**Figure 15:**
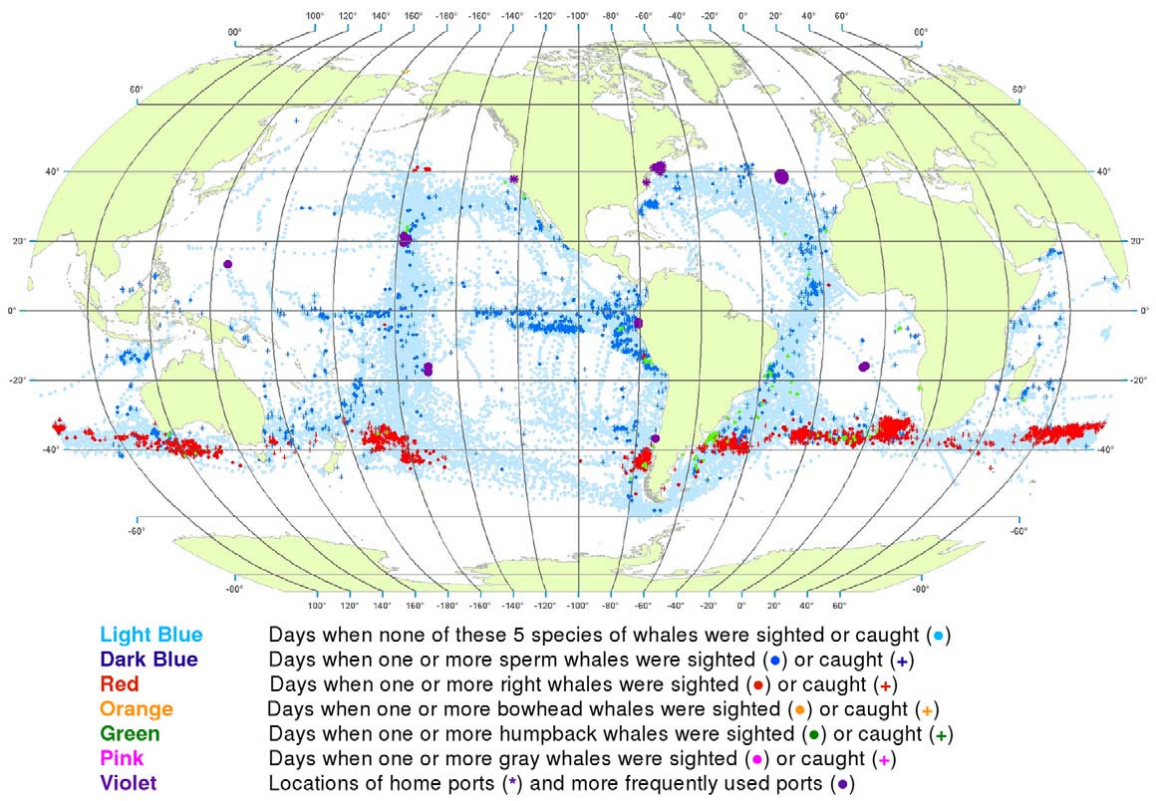
November observations of whales. The data were extracted from a sample of American whaling logbooks for voyages departing between 1780 and 1920. Days with no whale observations and days with observations of sperm, right, bowhead, humpback, and gray whales and locations of key ports were distinguished by the colors indicated.

Portions of the monthly maps sometimes suggest seasonal migrations. For example, female right whales are known today to occupy specific bays for calving once every three years. The calving bays used by southern right whales along the eastern coast of New Zealand in the winter [23] are suggested by observations from May through August (Figures 9, 10, 11, and 12). Beginning in September, they appear to have followed an arc, first moving northeastward (Figure 12), then eastward in October (Figure 14), then southeastward in November through February (Figures 15, 4, 5, 6) and finally westward toward the coast of New Zealand by April (Figure 8) [24].

Similar patterns of seasonal offshore and inshore movements of southern right whales are evident on a larger scale. The existence of winter calving and calf-rearing areas along the southern coast of Australia, the western and eastern coasts of South America, and the southern coast of Africa are suggested by the persistent, albeit few, observations in the months of May through August (Figures 9, 10, 11, and 12). Even during those months, however, there were observations well offshore, especially south and east of Africa, suggesting that some right whales did not move into areas close to the continental landmasses. After August, observations of southern right whales were spread out across open ocean areas around the globe, trending farther south in the spring and summer, i.e., from September to February (Figures 13, 14, 15, 4, 5, and 6). Considering that at that time of the year, right whales were observed fairly often in latitudes near the southern limit of whaling activity, it is not possible to infer the true southward extent of their movements from the whaling data. We note, however, that there was considerable other whaling activity south of the aggregations of observations around South America in all months and around New Zealand from November through February (Figures 15, 4, 5, and 6), suggesting that southern right whales did not migrate much farther south in those areas.

Sperm whales were observed primarily from February through April (Figures 6, 7, and 8) in the South Atlantic, especially along South America, and from May through September (Figures 9, 10, 11, 12, and 13) in the central North Atlantic. In contrast, sperm whales were observed in the western Indian Ocean off southern Africa and in the eastern Indian Ocean off Australia in all months, but most observations were made from March through June (Figures 7, 8, 9, and 10).

The monthly maps also reveal the timing of coastal calving and breeding activity of humpback whales and gray whales. American whalemen located winter humpback whale calving and breeding areas in all oceans (see Figures 9, 10, 11, 12, and 13 for the southern hemisphere and 4–10 for the northern hemisphere). In the equatorial Pacific along the west coast of South and Central America, humpback whales were observed year-round, likely representing separate northern and southern populations using similar grounds in their respective calving and breeding seasons [25]. The whalemen exploited the gray whale calf-rearing and breeding grounds along the Pacific coast of Mexico, and particularly the lagoons of Baja California, primarily from January through March (Figures 5, 6, and 7) [26].

### Changes Over Time

**Figure 16:**
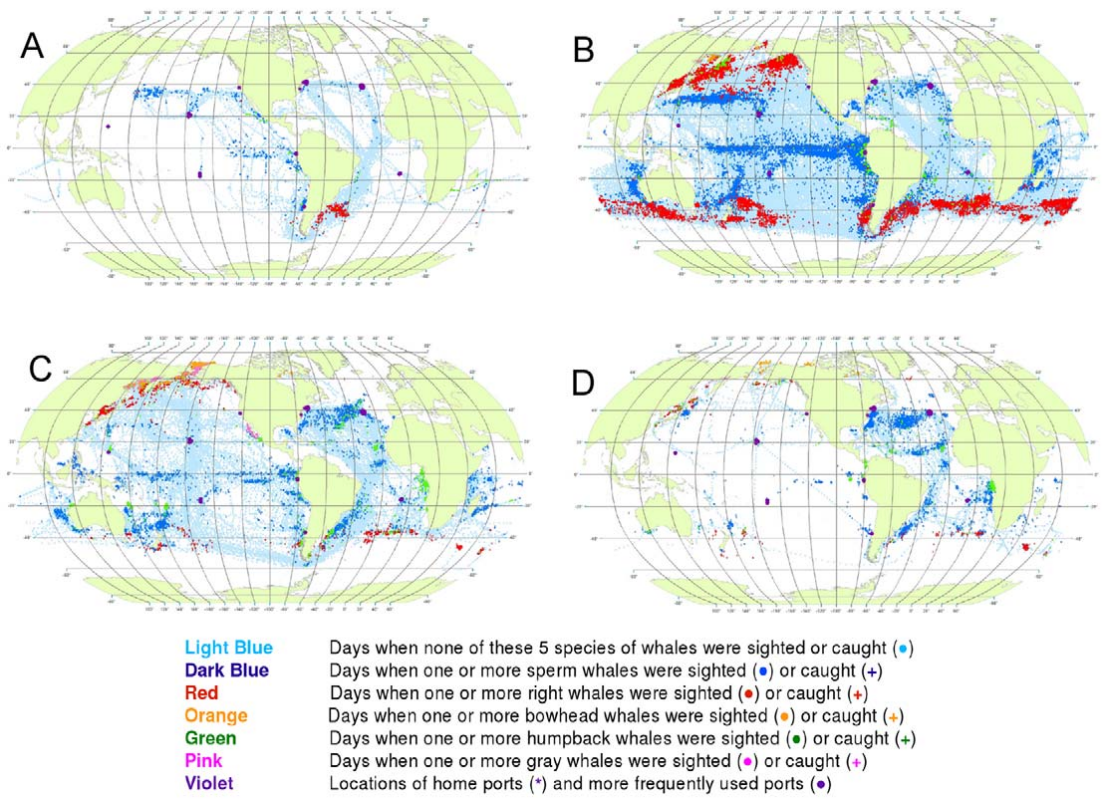
All observations of whales by time period. Daily locations of whaling vessels in: 1780–1824 (A), 1825–1849 (B), 1850–1874 (C), 1875–1920 (D). The data were extracted from a sample of American whaling logbooks for voyages departing between 1780 and 1920. Days with no whale observations and days with observations of sperm, right, bowhead, humpback, and gray whales and locations of key ports were distinguished by the colors indicated. The time periods were selected to best reflect the similarities in distribution patterns within time periods.

American whalemen expanded their reach out of the Atlantic and into the Pacific and Indian Oceans, depleting population after population of seven species of whales. Within each ocean and for each species, the spatial changes primarily reflect the continual search for new grounds as the regional abundance of whales declined, resulting in older whaling grounds being abandoned and new grounds being discovered [12]. The course of this expansion and exploitation can be seen by displaying the encounter data in four successive time periods (Figure 16).

1780–1824: Vessels from New England ports traversed the North Atlantic eastward and then southward (Figure 16A). American whaling was confined almost entirely to the Atlantic Ocean until 1792, and then expanded into the Pacific, apparently because of the ready availability of sperm whales there [27]. Right, sperm, and humpback whales were observed in the southwestern Atlantic, and sperm and humpback whales were observed in the eastern Pacific as far north as roughly 30°N.

1825–1849: American whaling expanded irregularly, to the west and north across the Pacific and into the Okhotsk and Bering seas, to the east across the South Atlantic, to the north in the western Indian Ocean, and to the east across the southern Indian Ocean (Figure 16B).

1850–1874: American whaling vessels continued to frequent most of the areas visited previously but with few observations of sperm whales in the North Pacific or right whales in the South Pacific, South Atlantic, and Indian oceans (Figure 16C). Observations of sperm whales were reported in the North Atlantic and of bowhead whales and gray whales in the North Pacific.

1875–1920: American whaling continued to decline in numbers of voyages and in landings [28], [29] as it contracted back into the Atlantic (Figure 16D). There the focus continued to be primarily on sperm whales. Only a few observations of right whales were made, and those were in around 40°S latitude. Humpback whales continued to be targeted on calving and breeding grounds in the southern North Atlantic and in the South Atlantic along the African coast. There was whaling in several coastal areas of the Pacific and western Indian oceans, some of which had not been exploited to a significant degree previously, for example the Gulf of Panama where humpback whales were available year-round.

The dominant feature of American whaling under sail was spatial expansion followed by contraction. Expansion reflects exploration and discovery of new concentrations of catchable, commercially valuable whales, whereas contraction reflects the exhaustion and abandonment of those areas of whale concentration and the declining demand for whale oil. One whaling historian, writing of the fishery for gray whales in the eastern North Pacific [30], characterized the pattern as an *initial* period, a *bonanza* period, and a *declining* period (in that instance spanning merely three decades from discovery to closure, 1845–1874).

This pattern can be discerned in our maps showing that gray whales were observed only between 1850 and 1873, when the fishery for them collapsed [26]. Similarly, there were few reports of sperm whales in the North Pacific after 1850 (compare Figure 16B and 16C) [7] despite the fact that whalemen continued to traverse formerly productive sperm whaling grounds as they searched for North Pacific right whales and bowhead whales. Also, the gradual decline in right whale observations (compare Figures 16B, 16C and 16D) [9], [15] and the decline in bowhead whale observations (Figures 16C and 16D) [8] indicate the probable depletion of these two species in the North Pacific and Western Arctic.

## Discussion

The observations in American whaling logbooks of sperm, right, bowhead, gray, and humpback whales that were the primary targets provide information on 19^th^ century distribution patterns that is available in no other way. Because the maps presented here include information about where whales were seen as well as where they were not, they allow us to infer some of the major features of the historical distributions of the seven targeted species of whales. This includes the low-latitude occurrence of sperm whales year-round, much of the high-latitude summer feeding range of all five groups, at least in the North Pacific and Western Arctic, and some of the winter calving and breeding areas of right, humpback, and gray whales. In addition, the maps indicate shifts in whaling effort through time, and many if not most of these shifts were likely due to local or regional depletion of the whale stocks. For example, after 1850 sperm whales were not regularly observed along the formerly productive 30°N latitude line in the North Pacific.

The data also reveal seasonal patterns, some of which are evident at a quarterly scale (Figure 3) and others at a monthly scale (Figures 4, 5, 6, 7, 8, 9, 10, 11, 12, 13, 14, and 15). To exploit the data fully in this regard, it is useful to examine the plots at higher resolution. High resolution versions of Figure 1 and Figures 4, 5, 6, 7, 8, 9, 10, 11, 12, 13, 14, and 15 are included as supporting information (Figures S1, S2, S3, S4, S5, S6, S7, S8, S9, S10, S11, S12, and S13). For example, using graphics manipulation software we extracted a relatively small region in the western North Pacific (from 20°N to 60°N latitude and 140°E to 180° longitude) for the months of February through August (Figures S4 S5, S6, S7, S8, S9, and S10). The resulting more detailed maps are shown in Figures 17, 18, 19, 20, 21, 22, and 23.

**Figure 17:**
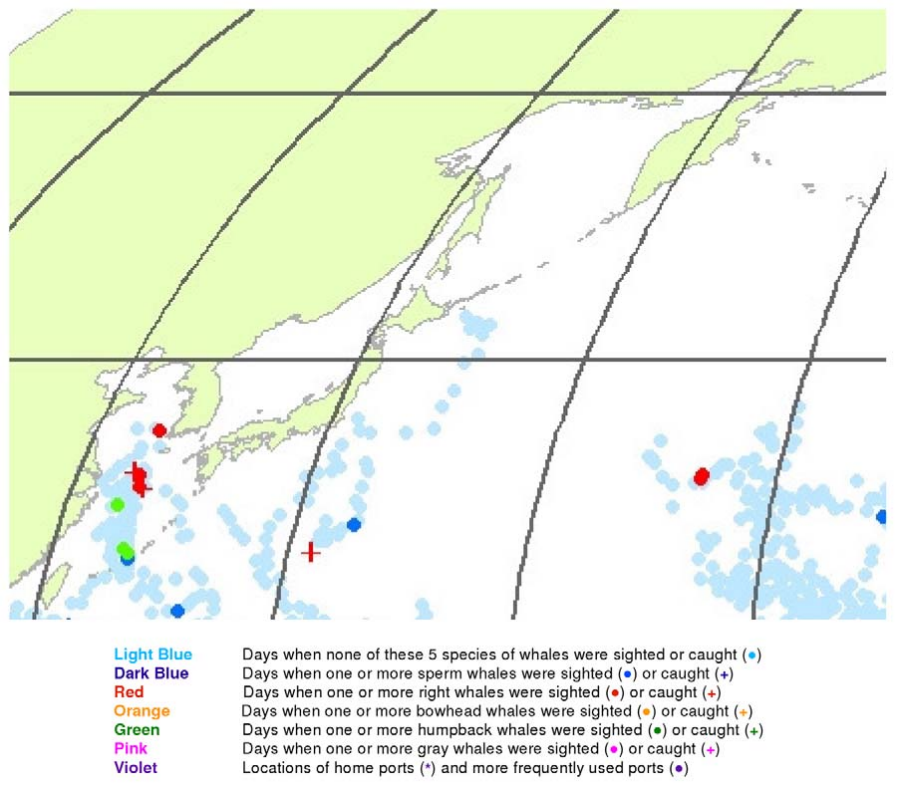
February observations of whales in the northwestern North Pacific. The data were extracted from a sample of American whaling logbooks for voyages departing between 1780 and 1920. Days with no whale observations and days with observations of sperm, right, bowhead, humpback, and gray whales and locations of key ports were distinguished by the colors indicated.

**Figure 18:**
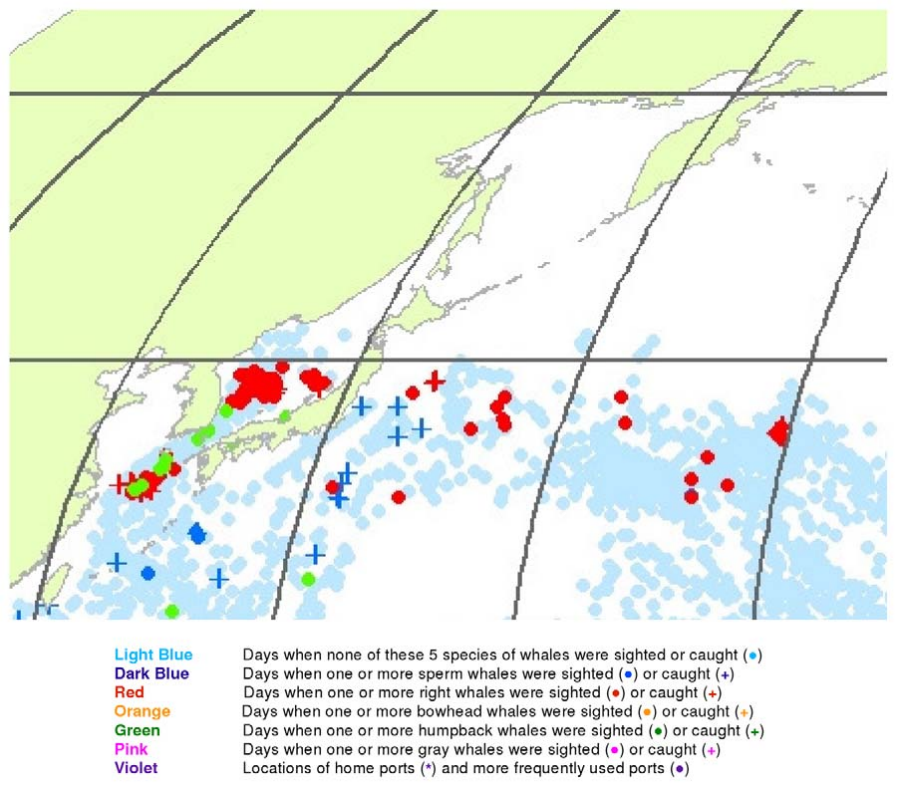
March observations of whales in the northwestern North Pacific. The data were extracted from a sample of American whaling logbooks for voyages departing between 1780 and 1920. Days with no whale observations and days with observations of sperm, right, bowhead, humpback, and gray whales and locations of key ports were distinguished by the colors indicated.

**Figure 19:**
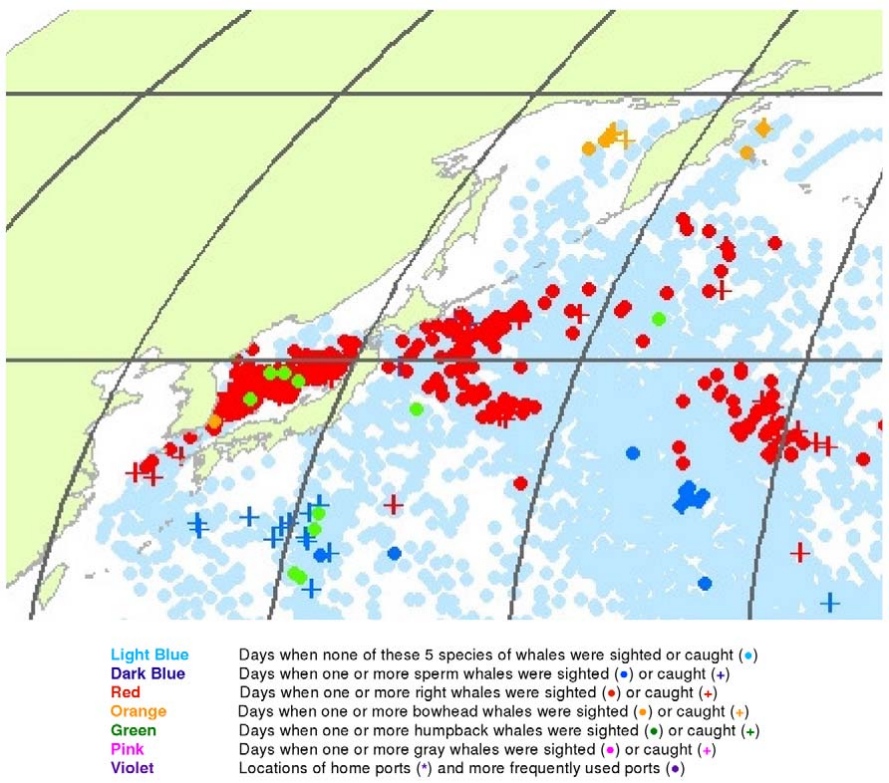
April observations of whales in the northwestern North Pacific. The data were extracted from a sample of American whaling logbooks for voyages departing between 1780 and 1920. Days with no whale observations and days with observations of sperm, right, bowhead, humpback, and gray whales and locations of key ports were distinguished by the colors indicated.

**Figure 20:**
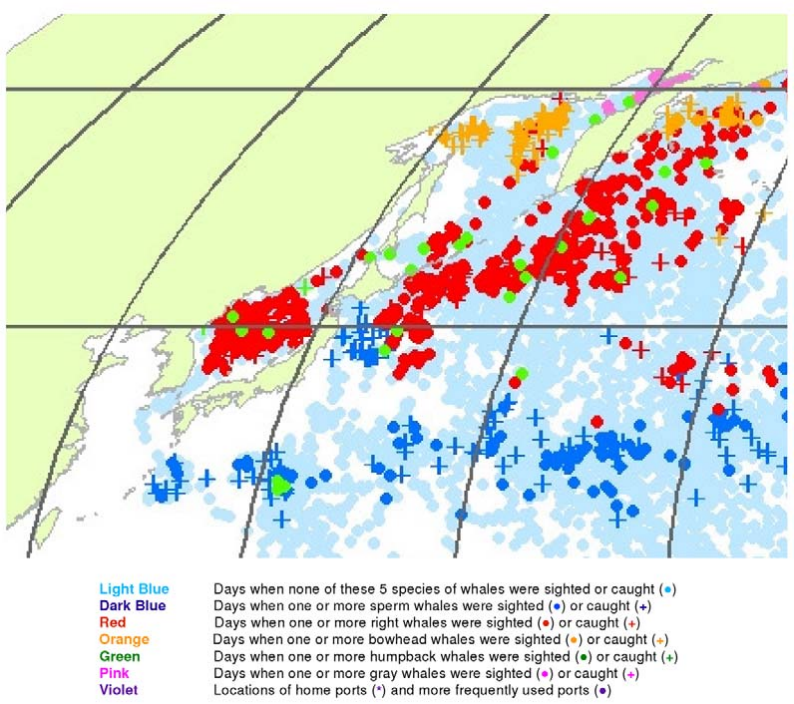
May observations of whales in the northwestern North Pacific. The data were extracted from a sample of American whaling logbooks for voyages departing between 1780 and 1920. Days with no whale observations and days with observations of sperm, right, bowhead, humpback, and gray whales and locations of key ports were distinguished by the colors indicated.

**Figure 21:**
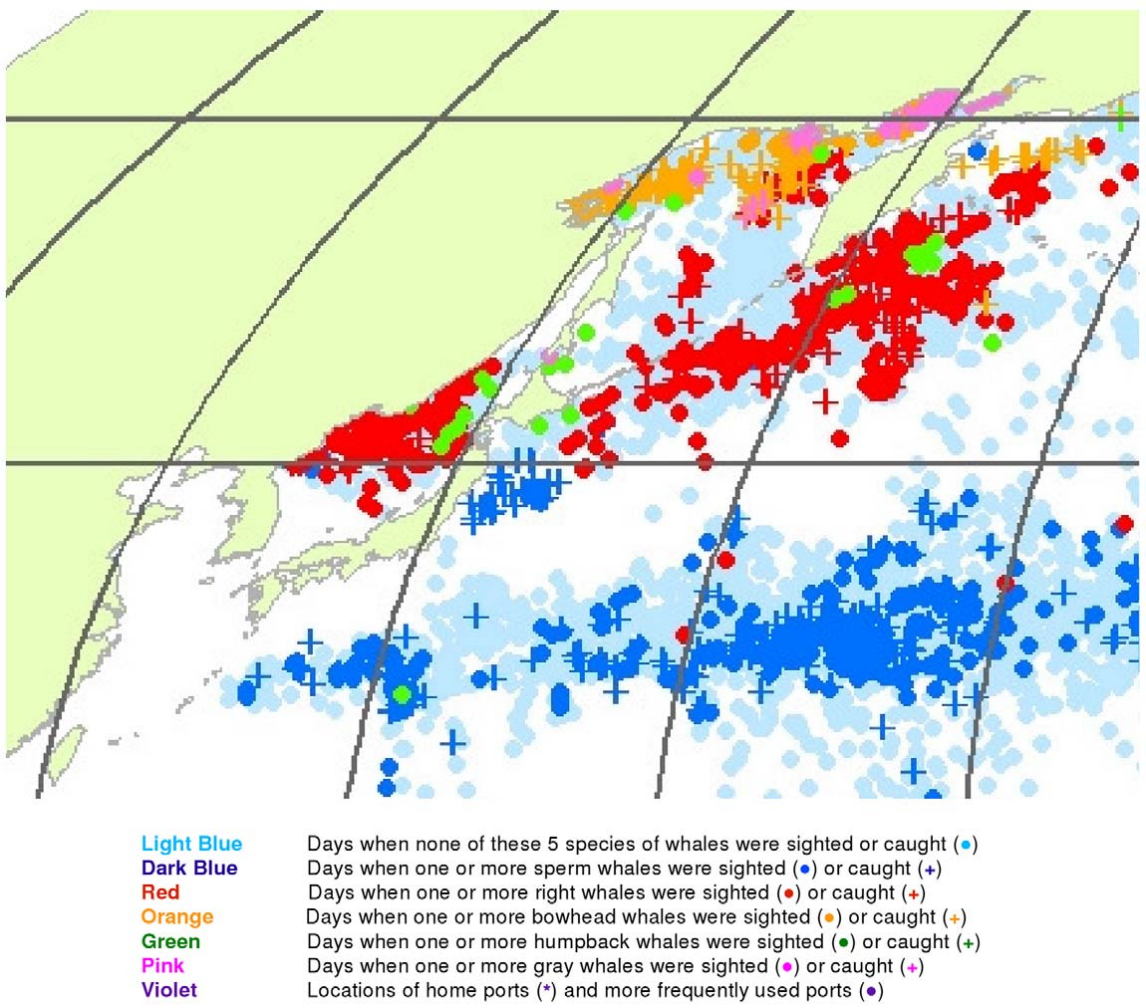
June observations of whales in the northwestern North Pacific. The data were extracted from a sample of American whaling logbooks for voyages departing between 1780 and 1920. Days with no whale observations and days with observations of sperm, right, bowhead, humpback, and gray whales and locations of key ports were distinguished by the colors indicated.

**Figure 22:**
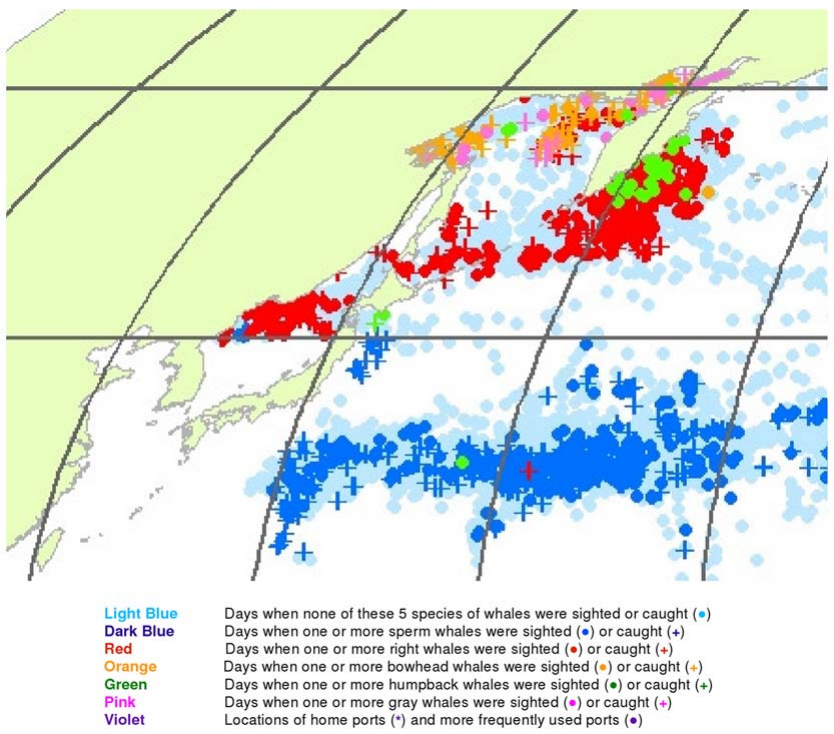
July observations of whales in the northwestern North Pacific. The data were extracted from a sample of American whaling logbooks for voyages departing between 1780 and 1920. Days with no whale observations and days with observations of sperm, right, bowhead, humpback, and gray whales and locations of key ports were distinguished by the colors indicated.

**Figure 23:**
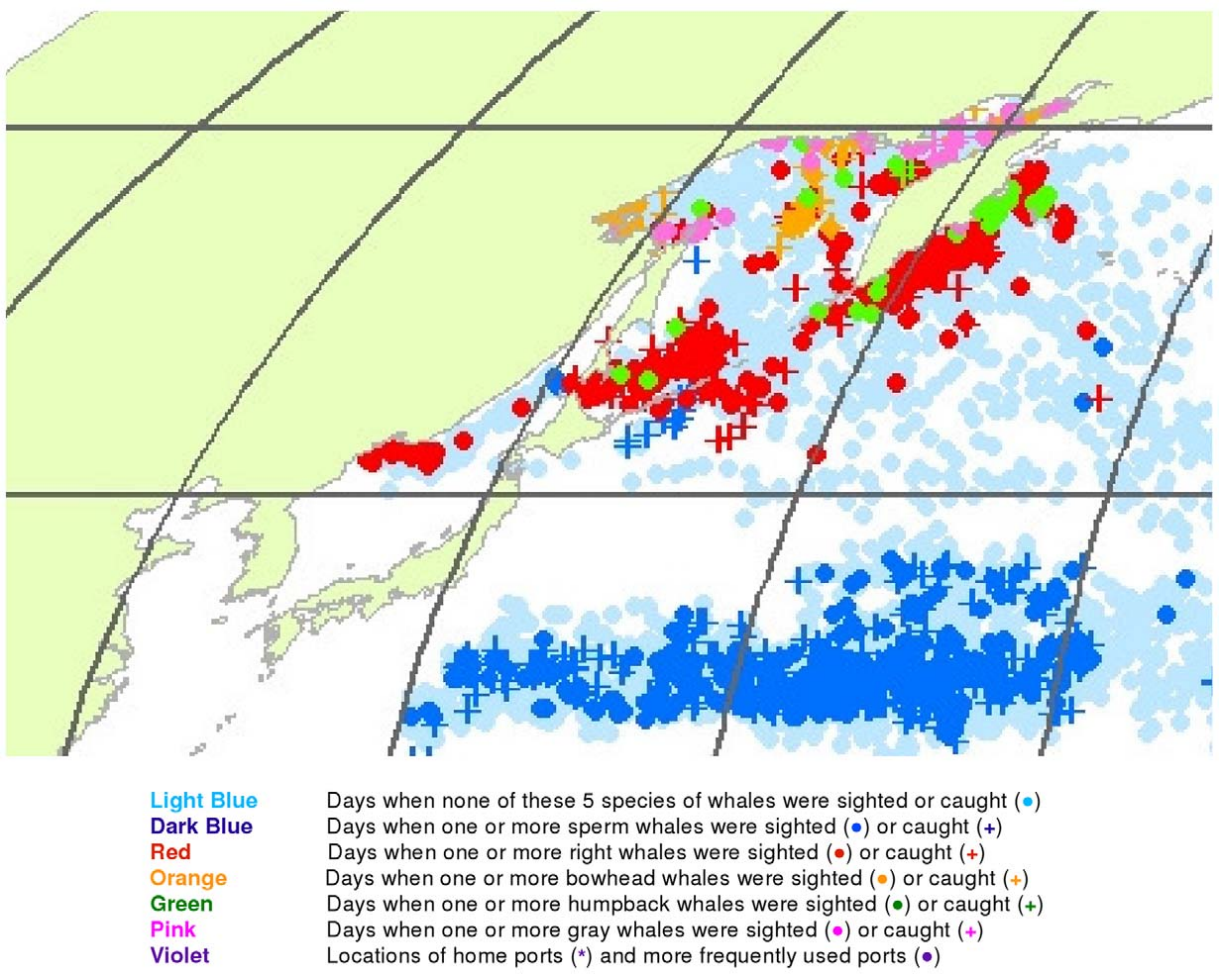
August observations of whales in the northwestern North Pacific. The data were extracted from a sample of American whaling logbooks for voyages departing between 1780 and 1920. Days with no whale observations and days with observations of sperm, right, bowhead, humpback, and gray whales and locations of key ports were distinguished by the colors indicated.

The light blue points in Figure 17 shows that American whalemen began searching this region by February, focusing on waters southwest of Japan in the East China Sea and on waters southeast of Japan, especially the seamounts along140°E longitude. At this time of the year they primarily encountered right whales and humpback whales in the East China Sea, but they saw few whales between there and 180° longitude. Humpbacks were observed in March and April in the Sea of Japan and right whales there and further east to 180° longitude between 30° and 50°N latitude (Figures 18 and 19). In contrast, sperm whales were observed primarily nearer Japan in February and March, and further east only beginning in April (Figure 19). After April, whales were observed mainly in two roughly longitudinal bands, with sperm whales encountered along 30°N latitude and right whales and humpback whales seen from Vladivostok in the Sea of Japan to the east coast of Kamchatka primarily north of 40°N (Figures 20, 21, 22, and 23). The fine-scale data from February to May suggest seasonal movements of right whales and humpback whales from the Asian coastline to the north and east. They also suggest movements of sperm whales into areas between 150°E and 170°E, coming either eastward from south of Japan or westward or northward from the central or southern North Pacific.

As mentioned earlier, other whaling operations, both vessel-based and shore-based, were carried out in the 19^th^ century [4]. There was considerable overlap in the grounds visited by the American whalemen and those used by shore whaling operations and offshore fleets of other nations. However, it must be acknowledged that such overlap was not complete and therefore the depiction of patterns in this paper would have differed, at least somewhat, if we had attempted to include all of the available information on those other operations.

We recognize that data obtained from whaling logbooks are fraught with uncertainties and difficulties of interpretation. Many of the limitations are discussed further below under Materials and Methods, but one point worth raising here is the question of how reliable the whalemen were as data recorders. In particular, did they record observations of whales accurately and consistently in the logbooks, such that the data extracted from the logbooks can be relied upon as representative of conditions at the time? In earlier work [31], we found that at least the catch data in the logbooks were generally reliable, leading us to conclude in [4] (page 94) that the “clear evidence of data manipulation and falsification in the twentieth-century has created what may be an unwarranted degree of skepticism toward earlier primary sources of whaling data.” We have no reason to believe the whalemen would have refrained from recording whale observations for strategic purposes, but there is nevertheless great variability in the level of detail provided in the logbooks (as well as variability in legibility, preservation, etc.). We attribute this variability to differences among the whalemen themselves in terms of literacy, experience, and interests, or perhaps more importantly, to the different priorities of owners, agents, and masters who prescribed what types of information should be kept in the logs. Non-target whale species were often recorded, including “finbacks” (probably most often fin whales, *Balaenoptera physalus*, but also presumably sei and Bryde’s whales, *B. borealis* and *B. edeni/brydei*, respectively), as were humpback whales and gray whales in areas and at times when no effort was made to pursue them, for example prior to 1850 [22] (Figure 16B).

**Figure 24:**
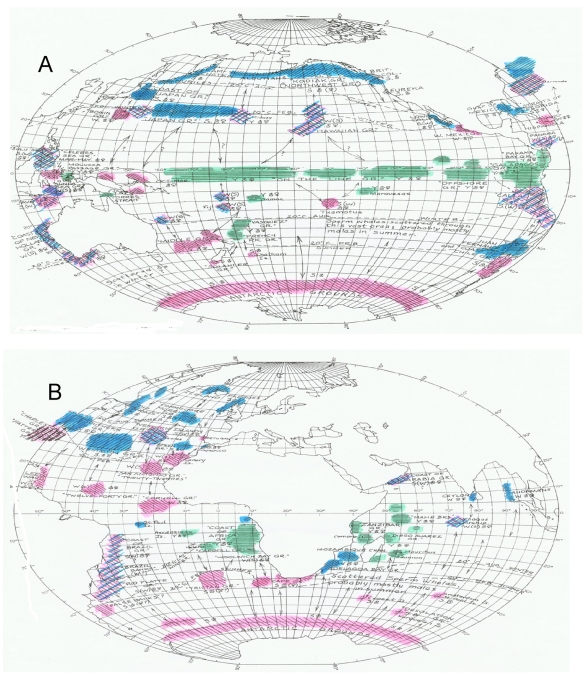
Raymond Gilmore’s 1959 world map of the distribution of sperm whales. Distribution of sperm whales in the Pacific Ocean (A) and in the Indian and Atlantic oceans (B). This map was modified by John Bannister [13] from maps prepared by Raymond Gilmore in 1959 [32]. Gilmore’s sources included Townsend’s 1935 charts A and B [11] and Gilmore’s own tabulation of modern steam whaling from 1865 to 1958. Shown are whaling grounds (marked by “GR”), principal seasons of the catches annotated by letters (summer="S”, winter="W”, year around="Y”) and by colors and hatching patterns (blue & left to right hatching = northern summer and southern winter, pink & right to left hatching  =  northern winter and southern summer, green & transverse hatching = year around), predominant sex of the catches (female = ♀, male = ♂), possible migration routes (marked by arrows), and 20°C surface isotherms in February and August (dashed lines).

However, it has been noted repeatedly that there is one anomaly in the logbook records that creates persistent doubts about their reliability. Although in many parts of the world, e.g. around New Zealand and along the east coast of South America (Figure 5 and 6), sperm whales and right whales were mentioned in the logs as being in close spatial and temporal proximity, this was not the case in the right whaling grounds north of 40°N in the North Pacific (compare Figures 2A and 2B), a region where 20^th^ century whalemen later took many sperm whales (Figure 24), raising the question of why 19^th^ century whalemen reported few there [31].

Another concern is that at least a few whaling grounds identified by other authors are not apparent on our maps of logbook data. For example, one map of the 19^th^ century whaling grounds shows a sperm whale ground along 30°S latitude from Peru westward to 170°W longitude [12]. That area is annotated on another more recent map of the global distribution of sperm whales and sperm whaling grounds as “Sperm whales scattered through this vast area, probably mostly males in summer” [32] (see Figure 24). There is no obvious explanation as to why this putative ground does not show up on our maps; vessels visited the area in all seasons (Figure 3).

In conclusion, we consider the American logbook data, as illustrated on the maps presented here, to be informative for understanding the historical development of the American offshore whale fishery as well as the global distribution of the seven species of whales targeted during the 19^th^ and early 20^th^ centuries. It is important to recognize that some of the whale populations exploited in the 19^th^ century are still far below their pre-whaling abundance; in some areas of formerly high-density occurrence, the animals are now absent or rare. Recolonization or recovery has often been hindered by 20^th^ century whaling, some of it illegal and poorly documented. At least one species, the North Atlantic right whale, is currently threatened by ship strikes and entanglement in fishing gear [33], [34]. The significance of those threats for other species as well as the possible threats of environmental change, ocean pollution, and other factors remain largely speculative.

## Materials and Methods

**Figure 25:**
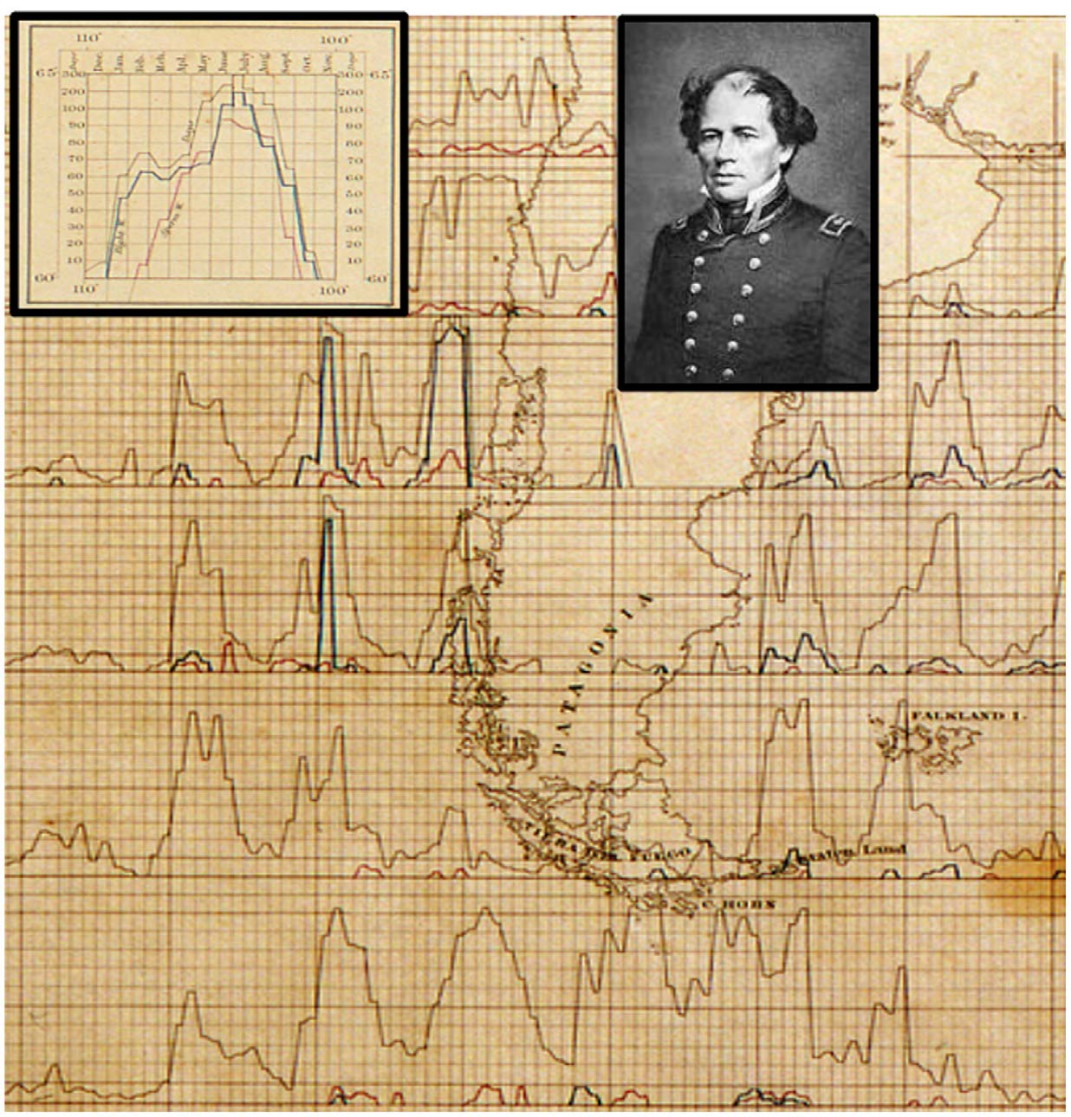
Maury’s 1853 whale chart around southern South America. Part of Maury’s 1853 whale chart summarizing catches and sightings of sperm and right whales along both the Atlantic and Pacific Ocean coastlines of South America south of 30°S, between 55°W and 90°W. Shown are 6 latitudinal and 7 longitudinal blocks of 5° of latitude by 5° of longitude. An inset of a diagrammatic single block illustrates the form of the data, which included the monthly number of days on which right and sperm whales were reported and the number of vessel-days at sea. Months are depicted from left to right horizontally, from December through November. For each month, the height of the lighter black line denotes number of vessel-days, and the height of the blue and red lines denotes the number of right whales and sperm whales encountered, respectively, according to a non-linear scale. Also shown is a photograph of LCDR Matthew Fontaine Maury.

The data set used here includes data collected in the two earlier studies by Maury [10] and Townsend [11] (both described briefly above) as well as data extracted from logbooks specifically for our present purposes. In the 1840s Maury began studying logbooks from naval, shipping, and whaling vessels and assembled a database of daily locations, weather reports, and whale observations (both sightings and catches). From these data, he created the first quantitatively grounded description of sperm whale and right whale distribution in the form of a series of graphs. Each graph summarized data for an area covering 5° of latitude and 5° of longitude, and included the monthly number of days on which sperm whales and right whales were reported in the logbooks as having been observed, along with the total the number of vessel-days at sea. These graphs were displayed on large-format maps of different regions of the world, which Maury referred to as “whale charts” [10], [35]. Figure 25, cut away from one of Maury’s maps, shows the coastlines of South America. The presence of both right whales and sperm whales near both the Pacific and Atlantic coasts between 40° and 50°S is apparent, and few whales were shown further west of South America.

**Figure 26:**
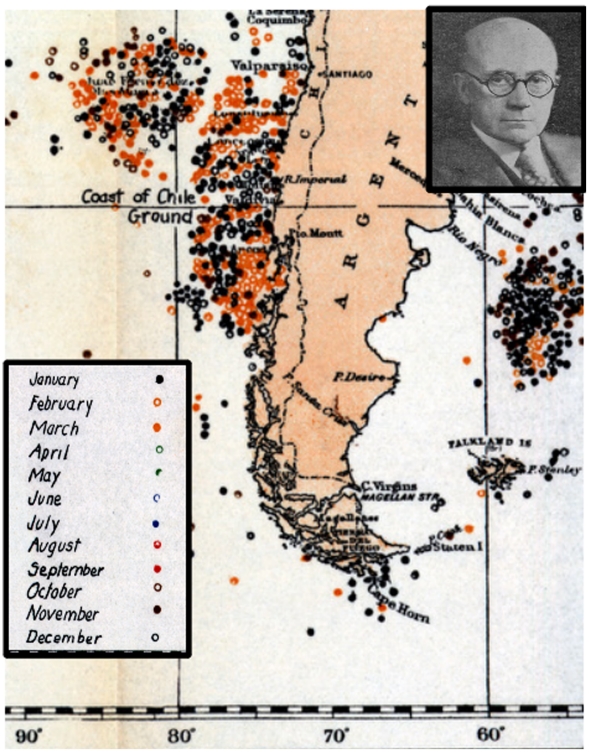
Townsend’s 1935 right whale chart around southern South America. Part of Townsend’s 1935 whale chart showing the Atlantic and Pacific Ocean coastlines of South America south of 30°S, between 55°W and 90°W, and the locations of observations (catches) of right whales, with colors indicating months as shown in the inset. Also shown is a photograph of Charles Haskins Townsend.

Almost a century later, well after the sailing era of American whaling had ended and the era of “modern” whaling had begun, Townsend applied a simpler, less labor-intensive method of data collection to a similar task. He recorded only the locations of the vessels on days when whales were actually caught, ignoring all other days whether whales were sighted or not. From these data, he created a series of maps, also referred to as “whale charts,” showing, for each species, the reported or interpolated locations of vessels on days with catches. Figure 26, cut away from one of Townsend’s maps, shows the location of right whales caught along both coastlines of South America, color coded by month.

A major difference between the Maury and Townsend studies was that Maury recorded and tried to convey information on where the whalemen searched, noting, “it is important to have a complete abstract for every day at sea, that we may know whether they find fish or not…” [35]. This means his maps show not only where whales were observed, but also where the whalemen searched for them but reported none. In contrast, Townsend’s maps show only positive information, i.e., the locations where whales were caught, and in what months.

Although both Maury’s and Townsend’s maps include information on a monthly scale, the patterns are difficult to discern and interpret without considerable effort on the reader’s part. In the case of Maury’s maps, determining monthly trends requires close inspection of many line graphs. Similarly, in the case of Townsend’s maps, the technical constraints of color mixing and printing in the 1930s limited the resolution and precision of the color-coded dot scheme. Furthermore, although it is tempting to look for changes in whale distribution that might have occurred between the Maury period (ca. pre-1850) and the latter half of the 19th century which is included in Townsend’s depictions, the desired comparisons are not possible. Not only do the two sets of maps differ in terms of format, scope, and approach (e.g. compare Figures 25 and 26), but also both sets of maps have inaccuracies and idiosyncrasies that confound any comparative analysis [9], [36]–[39].

Here we extend the approach taken by Maury and Townsend by presenting maps of the daily locations of American whaling vessels to illustrate the historical spatial and temporal distribution of both the whaling fleet and the seven species of whales being sought by that fleet, drawing on the three data sources described above and integrating the data using modern methods of computer graphics. These maps enable new interpretations and comparisons of the logbook data that were not possible from the Maury and Townsend efforts.

### Logbook Data

Maury’s maps were based on daily vessel locations extracted from logbooks and recorded on data sheets labeled “Maury Abstracts” [10], [35]. The abstract of a given voyage included daily records of vessel location, weather, presence of whales, and other observations. We located 88 microfilms containing the 355 volumes of Maury Abstracts known to be extant (representing roughly two-thirds of the 533 volumes prepared), including merchant, naval, and whaling voyages. We also located computer files of data extracted by NOAA from 87 of the 88 Maury Abstracts, including daily positions and weather observations. We augmented those computer files with information from the Maury Abstracts on whales sighted and caught by examining the microfilms. We were able to identify daily data for more than 670 whaling voyages between 1797 and 1855. We matched Maury’s voyages to a list of all known American whaling voyages, with each voyage assigned a unique identification number [29].

Maury did not describe how his sample of logbooks was selected, but we know that the two assistants who extracted most of the data for his project were based in major whaling ports. One of them, Daniel McKenzie, had been master of eight whaling voyages between 1818 and 1846. Some published correspondence from him to Maury [35] suggests that McKenzie had been actively involved in the whaling community and had read logbooks made available to him by whaling masters, vessel owners, or agents. However, we do not know whether Maury or his assistants applied any selection criteria to the logbooks they sought or otherwise had available to them.

Townsend’s maps were based on data extracted from whaling logbooks and recorded on unpublished data sheets, including vessel name, date, location, and species taken. These data sheets were available to us only for voyages by vessels with names beginning with the letters A through J [37]. Given the efforts by various interested parties over the last few decades to locate the rest of Townsend’s worksheets, we have no reason to believe they are extant. We digitized the available worksheets, and matched the voyages to the list of American whaling voyages and their respective unique identification numbers [29]. Townsend provided few clues as to how his source logbooks were selected but in his published account accompanying his maps, he included a comprehensive table showing vessels (listed alphabetically), voyage years, and catch by species for each voyage represented on his maps as well as lists of the institutional and individual owners of the logbooks [11].

As part of the Census of Marine Life [8] (CoML), we read a sample of voyage logbooks and journals in public collections [17], [29]. This sample was selected using the unique voyage identification numbers with the goal of achieving representative coverage of the fishery over time, taking due account of the coverage already provided by the Maury and Townsend samples. Logbooks were selected roughly randomly within decades, but preference was given to complete logbooks (i.e., those covering a voyage from start to finish) which regularly included daily latitude and longitude observations and the species of whales observed and/or hunted. For each voyage, we recorded daily information on vessel location, species of whales sighted, struck, or caught (landed), along with other information such as the yield of oil from individuals or groups of whales.

### Completeness of Logbook Data

We selected compatible fields from the Maury, Townsend, and CoML data sets and combined them, recognizing that the data sets differed in the collection protocols used and that for some voyages, the document read in one study was not the same as the one read in another study (such that, for example, a voyage might be represented by a partial log or journal in one case and a complete log or journal in the other). The combined data are from 1,458 logbook readings, as summarized in Table S1, where the voyage identity is shown based on a catalog of voyages [29].

Obvious temporal gaps are present in the data from some of the Maury and CoML samples, arising from the incompleteness of the logbooks (e.g., no entries for some days, no positions given on some days). In addition, in some instances, particularly the case with journals kept by individual whalemen, entries only started after the voyage was already underway, or the document was truncated before the voyage ended, or there were short-duration gaps in coverage from time to time, probably when the men were busy with whaling tasks, or long-duration gaps for unknown reasons. Also, Maury’s data often do not include entries for days when the vessel was north of the equator in the Atlantic, even though most logbooks contained entries for this region. Further, there are some temporal gaps in Maury’s data for seasons when whaling was occurring in bays, for example in right whale calving bays along the southwestern coast of Australia in the winter. It was not possible to identify gaps in the Townsend sample due to the absence of effort data, i.e., information on days when the vessel was at sea but no whales were taken. For some voyages, the total reported returns of sperm oil or baleen whale oil [29] were inconsistent with the numbers of whales recorded in Townsend’s catch data, suggesting that some of the logbooks used by Townsend and his assistant were incomplete or incompletely read.

Where daily entries for latitude or longitude (or both) were missing, we either interpolated the position from previous and subsequent positions or, if recognizable place names were given, assigned representative positions. In some situations, especially where the whaling operations were conducted in or near the same place (e.g. near an island or continental shoreline), all logbook entries without positions but with the same place-names were assigned identical representative positions.

### Accuracy of Logbook Data

Some logbooks were not very legible or were far from complete. In the CoML sample, we rejected logbooks that we judged to be poor in either way. We do not know if Maury or Townsend made similar selections. We used information about each voyage obtained from readily available non-logbook sources to supplement the logbook data [29], often including the official beginning and ending dates of the voyage (as compared to the beginning and ending dates in the logbook), announced destination(s), and whale product returns. Such information was compared with the logbook data to detect inconsistencies and errors.

The species was not recorded for some whales observed. This probably occurred when the whalemen themselves were unsure what they had seen, but it often also occurred when the keeper of the logbook apparently assumed that what they had seen would be obvious from the context (e.g. the vessel was on a well-known sperm whale ground so reference to a “whale” would obviously be to a sperm whale). For the Townsend and CoML data, even when the species of whale was not explicitly recorded on the worksheets, it was often possible to infer it by examining the surrounding events and descriptions. For Maury’s data, however, we found that sometimes even when the species was clearly recorded in the original logbook, it was not recorded as such by Maury’s logbook reader.

Further, it appears that log keepers did not always record all observations of whales in all areas. For example, we gained the impression from reading logs that whale observations were not made or recorded early in the outbound portions of some voyages and especially during return portions when the vessel could not store additional whale products (i.e., it was a full ship) and so had ceased whaling. Also, it is likely that some whales were not reported because the whalemen were in pursuit of other whales or otherwise preoccupied at the time. This probably applies especially to the less desirable whales like humpback whales or gray whales, which would have been of no interest to some whalemen, at least at particular times or in particular areas.

A related problem is that some species of whales had not yet been clearly distinguished from one another when they first became targets of American whaling. Specifically, bowhead whales in the Okhotsk and Bering seas were not routinely distinguished from right whales until the mid 19^th^ century, and even then the distinction was not always made consistently [35]. Thus, for example, right whales are shown in Figure 2B as occurring in and north of the Bering Strait. Some if not all of these were more likely bowhead whales, but we did not attempt to correct such identifications.

Accuracy of positions was addressed by scrutinizing the sequence of daily positions in the logbook data for gaps and inconsistencies that could have been caused by recording errors on the part of the log keeper or transcription errors on the part of the logbook reader.

For the Maury and CoML data, we detected such errors by comparing computed spherical distance between successive positions and examining (by eye) the tracks of the voyages with anomalously long distances traversed. We also identified errors by looking for triplets of successive days with apparently large movements away from and back to an area. Based on these examinations, we corrected some obvious errors by inspection of the data (e.g. incorrect recording of hemisphere, transposition of digits in the latitude or the longitude), and we corrected some less obvious errors by referring back to the original documents when possible. For the Townsend data, where positions were only recorded when a whale was killed, we compared the consistency of the positions with all place names recorded. We also examined the consistency of the positions recorded on successive or closely spaced days.

Using the Maury and CoML samples, the average daily spherical distance computed from successive logbook positions on the 438,652 days during which the logs indicated vessel movement was 122 km (standard deviation 110.0 km). The frequency of daily distances declined monotonically, with less than 0.08% of the observations being greater than 750 km. By comparison, the maximum recorded speed of the faster clipper ships in the 1800s was roughly 750 km per day [40]. This suggests that there remained at least a few undetected errors in the positions as recorded in the logbooks or as transcribed while reading them.

Upwardly biased single-day transit distances could occur through onboard errors in determining a vessel’s position, for example due to mathematical errors or occasional adjustments to chronometers. To evaluate this, we attempted to measure the combined effect of these factors by locating in the CoML data days when masters of two or more voyages “spoke” each other and where both logbooks included positions for that day. We located 32 such days, and the mean absolute difference between pairs of reported positions was 0.22° of latitude (standard deviation of 0.315 degrees) and 0.54° of longitude (standard deviation of 0.667 degrees). Routine errors of this magnitude would not account for the unreasonably large single-day transit distances in the data.

On the other hand, extenuating circumstances may have contributed to large single-day transits in some instances. For example, during the 1850–1854 voyage of the bark *Fortune* of New Bedford (unique voyage identification number 5040), logbook data suggested a single-day transit of over 1000 km, and examination of the data reveals no obvious errors. After exiting the Okhotsk Sea on 17 October south of “Paramouchir Island” (Paramushir Island) at 50°00′N, 155°05′E, *Fortune* began traveling southeast toward Hawaii. The log keeper noted on both 20 and 22 October that the vessel was in a hurricane. On the subsequent two days (23 and 24 October), the positions given in the log were 48°48′N, 157°40′E and 47°N, 171°15′E, respectively, giving a calculated transit distance of 1032 km. The log subsequently indicates several times that the vessel was leaking and by 18 November notes, “people getting better and washing bone.” *Fortune* arrived at Maui on 26 November 1853.

From these observations, we concluded that there were unexplained errors in the recorded positions in at least a small proportion of the logbook data. To minimize any possible effects of these anomalous positions on our maps, we omitted data points that implied daily movements greater than 750 km.

### Representativeness of Logbook Data

For four time periods, we compared the number of known American whaling voyages [29] and the number of voyages for which we had logbook data (Table S1), by the three sources and in total (Table 1). The Maury study covered primarily the first half of the 19th century while the Townsend and CoML studies covered the entire span of years. The CoML sample was more uniformly distributed in time than the Townsend sample, which included a higher proportion of voyages in the second half of the 19th century. Data were extracted from logbooks, either directly by our reading (CoML) or indirectly via the Maury Abstracts or Townsend worksheets, covering a total of 1,458 voyages, of which 1,381 were unique. The unique voyages constituted between 2.1 and 14.4% of all voyages known for each of the four periods, with an overall sampling fraction of 9.8% of all voyages (Table 1). The relatively low sampling rate of voyages departing between 1780 and 1824 (2.1%) and the expanding spatial distribution of American whaling between 1780 and 1849 (Figure 16A and 16 B), means that our maps may under-represent whaling grounds used only or mainly in early years.

**Table 1:** For four time periods, the number of American offshore whaling voyages that were conducted (All Voy) [29], the numbers of voyages in the CoML, Maury and Townsend samples, respectively, the number of unique voyages sampled (Unique Voy), and the percent of all voyages that were sampled.

Time Period	1780–1824	1825–1849	1850–1874	1875–1920	1780–1920
All Voy	2314	5465	4125	2184	14088
CoML sample	18	74	67	37	196
Maury sample	22	529	16	0	567
Townsend sample	10	236	275	174	695
Unique Voy	48	785	346	202	1381
% All Voy sampled	2.1	14.4	8.3	9.2	9.8
% NB Voy	22.5	31.3	40.6	32.8	32.8
% NB Voy sampled	4.4	21.2	14.7	20.4	16.8

Also shown are the percent of all voyages that sailed from New Bedford (%NB Voy) and the percent of those voyages that were sampled (%NB Voy sampled).

Among possible reasons for the uneven sampling rate through time is that logbooks from more recent voyages are more likely to have been preserved. This could cause voyages from ports that were important in the earlier portion of our study period to be under-represented simply because fewer logbooks from such ports are extant and available. Further, all three source studies were centered in New Bedford: that city replaced Nantucket, MA, as the most important port during the period covered by Maury’s data collection effort and institutions in New Bedford held a majority of the logbooks used in both the Townsend and CoML efforts.

Logbooks of voyages from New Bedford were sampled at a rate of 16.8% compared with the 9.8% overall rate (Table 1). This differential varied over the study period, being somewhat less for the 1825–1849 interval. The degree to which New Bedford voyages are representative of all American voyages is difficult to ascertain, but systematic differences between whaling grounds used by vessels from New Bedford and those used by vessels from other ports would reduce the representativeness of our maps in ways that are difficult to predict without additional detailed analysis.

### Mapping

We mapped all of the data from the 1,458 “voyages” (recognizing that there were some duplicates) because the completeness of the data differed among the three data sets. For example, Townsend recorded only catches while Maury and CoML also recorded sightings. For our purposes, any duplicated observations would be overlaid on the maps and thus they would not change the overall depiction of the distribution of vessels or whales.

We used the Arc-GIS program (Economic and Social Research Institute, www.esri.com) to plot the daily geographic positions of whaling vessels on global maps using a Robinson projection [41]. This projection balances equal-area and conformal projections, and was selected for its minimal distortion in the regions where most American whaling occurred while avoiding strong curvature of the meridian lines. We centered the maps at 100°W longitude, hence wrapping around at 80°W longitude. This centering was selected because positional information was relatively sparse at that longitude and only right whales were routinely reported in the southern Indian Ocean. The positions were shown as color-coded symbols to distinguish days when no whales were observed and when one or more of each of the seven main species were seen or taken (sperm, right, bowhead, humpback, and gray). Different symbols were used to distinguish sightings and catches and to show the locations of home ports and frequently used ports.

When vessels were reported at nearby or identical locations, the symbols became overlaid, making it difficult or impossible to distinguish them. We attempted to minimize this problem in cases where there were many identical positions, for example a bay where the positional information was given repeatedly in the logbook(s) as a single place-name, by randomly reassigning those positions within a “circle” of 1° latitude and longitude around the reported or assigned location.

This helped tease out information that otherwise would have been obscured, e.g. humpback whale wintering areas in the South Pacific (Figure 2C). However, it also occasionally resulted in misleading impressions. For example, it caused a few observations of humpback whales in the Gulf of Panama to be plotted in the Caribbean Sea at around 80°W longitude rather than where they belonged in the Pacific Ocean (Figure 2C).

Symbols for locations with no recorded whale observations and those with sightings or catches of different species were overlaid in a consistent order, beginning with vessel present but no whale observations, then sperm whales, right whales, bowhead whales, humpback whales, and finally gray whales. This ordering was intended to highlight the less frequently observed species over those more frequently observed. Land masses were added only after all of the symbols had been plotted; in this way, observations with unreasonable positions (i.e., on land) were covered over. The aggregate degree of overlay of effort and whale symbols can be judged from Figure 2.

## Supporting Information

Figure S1 **High resolution map of all observations of sperm, right, bowhead, gray, and humpback whales.** Daily locations of vessels were extracted from a sample of American whaling logbooks for voyages departing between 1780 and 1920. Days with no whale observations and days with observations of sperm, right, bowhead, humpback, and gray whales and locations of key ports were distinguished by the colors indicated.
(JPG)

Figure S2 **High resolution map of December observations of whales.** The data were extracted from a sample of American whaling logbooks for voyages departing between 1780 and 1920. Days with no whale observations and days with observations of sperm, right, bowhead, humpback, and gray whales and locations of key ports were distinguished by the colors indicated.
(JPG)

Figure S3 **High resolution map of January observations of whales.** The data were extracted from a sample of American whaling logbooks for voyages departing between 1780 and 1920. Days with no whale observations and days with observations of sperm, right, bowhead, humpback, and gray whales and locations of key ports were distinguished by the colors indicated.
(JPG)

Figure S4 **High resolution map of February observations of whales.** The data were extracted from a sample of American whaling logbooks for voyages departing between 1780 and 1920. Days with no whale observations and days with observations of sperm, right, bowhead, humpback, and gray whales and locations of key ports were distinguished by the colors indicated.
(JPG)

Figure S5 **High resolution map of March observations of whales.** The data were extracted from a sample of American whaling logbooks for voyages departing between 1780 and 1920. Days with no whale observations and days with observations of sperm, right, bowhead, humpback, and gray whales and locations of key ports were distinguished by the colors indicated.
(JPG)

Figure S6 **High resolution map of April observations of whales.** The data were extracted from a sample of American whaling logbooks for voyages departing between 1780 and 1920. Days with no whale observations and days with observations of sperm, right, bowhead, humpback, and gray whales and locations of key ports were distinguished by the colors indicated.
(JPG)

Figure S7 **High resolution map of May observations of whales.** The data were extracted from a sample of American whaling logbooks for voyages departing between 1780 and 1920. Days with no whale observations and days with observations of sperm, right, bowhead, humpback, and gray whales and locations of key ports were distinguished by the colors indicated.
(JPG)

Figure S8 **High resolution map of June observations of whales.** The data were extracted from a sample of American whaling logbooks for voyages departing between 1780 and 1920. Days with no whale observations and days with observations of sperm, right, bowhead, humpback, and gray whales and locations of key ports were distinguished by the colors indicated.
(JPG)

Figure S9 **High resolution map of July observations of whales.** The data were extracted from a sample of American whaling logbooks for voyages departing between 1780 and 1920. Days with no whale observations and days with observations of sperm, right, bowhead, humpback, and gray whales and locations of key ports were distinguished by the colors indicated.
(JPG)

Figure S10 **High resolution map of August observations of whales.** The data were extracted from a sample of American whaling logbooks for voyages departing between 1780 and 1920. Days with no whale observations and days with observations of sperm, right, bowhead, humpback, and gray whales and locations of key ports were distinguished by the colors indicated.
(JPG)

Figure S11 **High resolution map of September observations of whales.** The data were extracted from a sample of American whaling logbooks for voyages departing between 1780 and 1920. Days with no whale observations and days with observations of sperm, right, bowhead, humpback, and gray whales and locations of key ports were distinguished by the colors indicated.
(JPG)

Figure S12 **High resolution map of October observations of whales.** The data were extracted from a sample of American whaling logbooks for voyages departing between 1780 and 1920. Days with no whale observations and days with observations of sperm, right, bowhead, humpback, and gray whales and locations of key ports were distinguished by the colors indicated.
(JPG)

Figure S13 **High resolution map of November observations of whales.** The data were extracted from a sample of American whaling logbooks for voyages departing between 1780 and 1920. Days with no whale observations and days with observations of sperm, right, bowhead, humpback, and gray whales and locations of key ports were distinguished by the colors indicated.
(JPG)

Table S1 **List and description of the 1458 samples of whaling voyages.** List of 1458 whaling voyages (by 689 American vessels) that were “sampled” by reading logbooks, 1780-1920. Shown are vessel name (Vessel), vessel number (Ves), voyage number (Voy), departure year (DepYr), and arrival year (ArrYr) [29]. The sources of the data are given as Src: CoML = Census of Marine Life, Maury = Matthew Fontaine Maury, and Town = Charles Haskins Townsend. Also shown are the number of days on which no whale observations were recorded (NoObs), the total number of whale observations recorded (Obs), the number of observations of identified whales (Sperm, Right, Bowhead, Humpback, Gray), and the number of other or unidentified whales (Other). NoObs is zero for all data from Townsend as that study did not record days when no whales were taken. Some samples of whaling voyages did not include any whale observations, although they did record the location of the vessel on most days. In some cases data from logbooks for the same voyage were available from more than one of the sources. See text for details on the data collection protocols used by the three sources.
(XLS)
