# Supplementary figures and images for: Spatial and Seasonal Distribution of American Whaling and Whales in the Age of Sail
Source: PLoS One. 2012 Apr 27;7(4):e34905. doi: 10.1371/journal.pone.0034905 (PMC3338773; doi:10.1371/journal.pone.0034905)

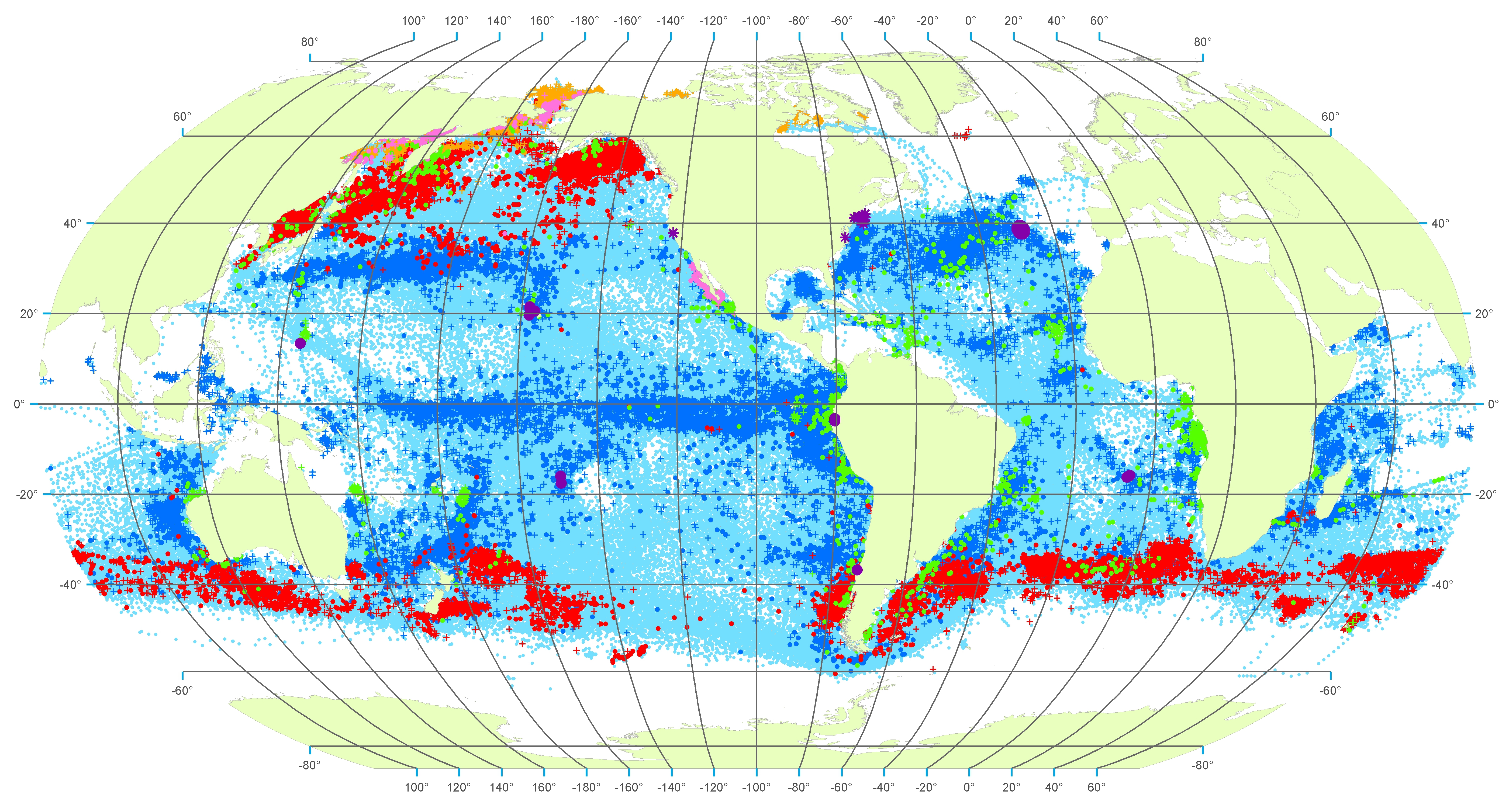

Supplement: Figure S1 — High resolution map of all observations of sperm, right, bowhead, gray, and humpback whales. Daily locations of vessels were extracted from a sample of American whaling logbooks for voyages departing between 1780 and 1920. Days with no whale observations and days with observations of sperm, right, bowhead, humpback, and gray whales and locations of key ports were distinguished by the colors indicated. (JPG) [file pone.0034905.s001.jpg]

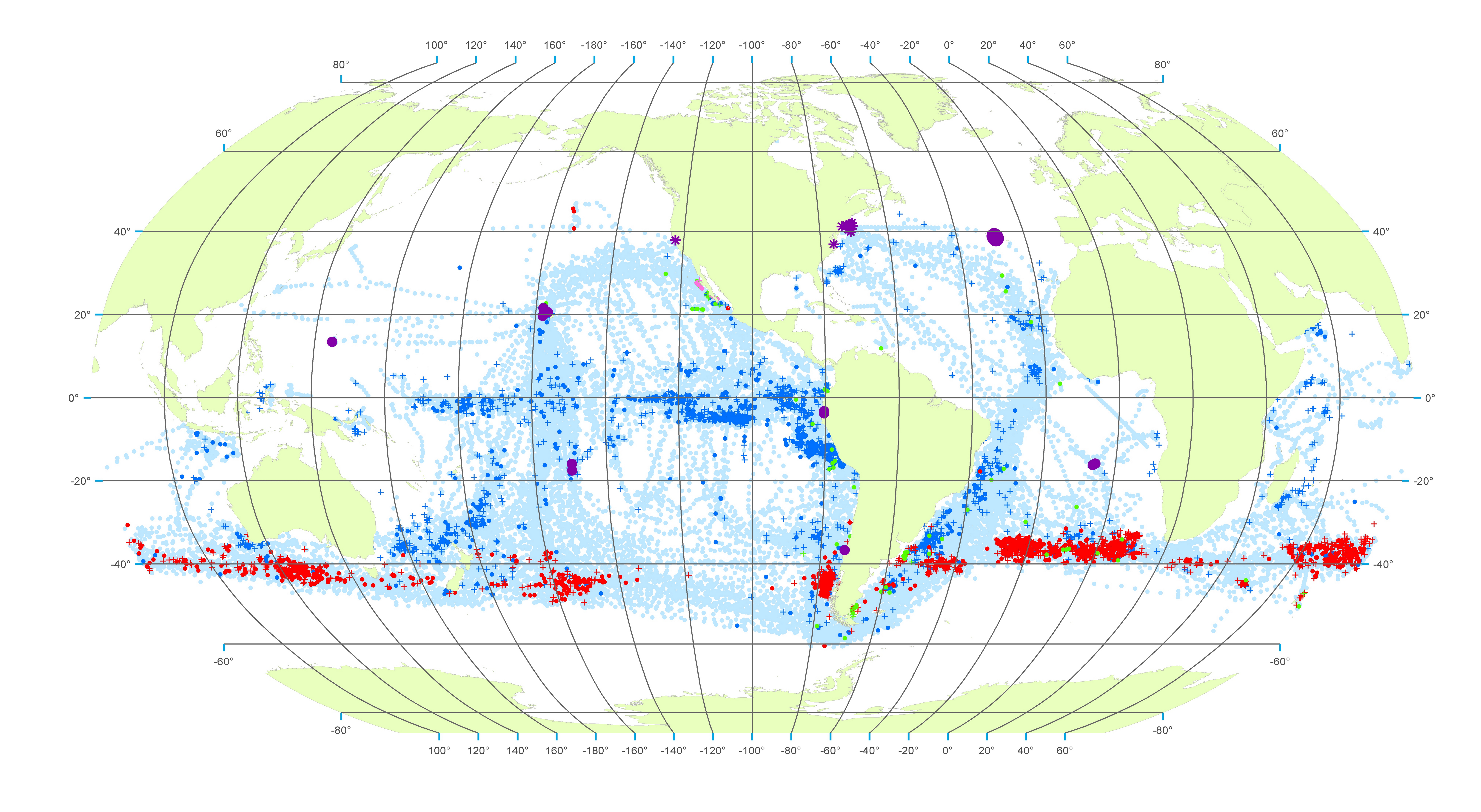

Supplement: Figure S2 — High resolution map of December observations of whales. The data were extracted from a sample of American whaling logbooks for voyages departing between 1780 and 1920. Days with no whale observations and days with observations of sperm, right, bowhead, humpback, and gray whales and locations of key ports were distinguished by the colors indicated. (JPG) [file pone.0034905.s002.jpg]

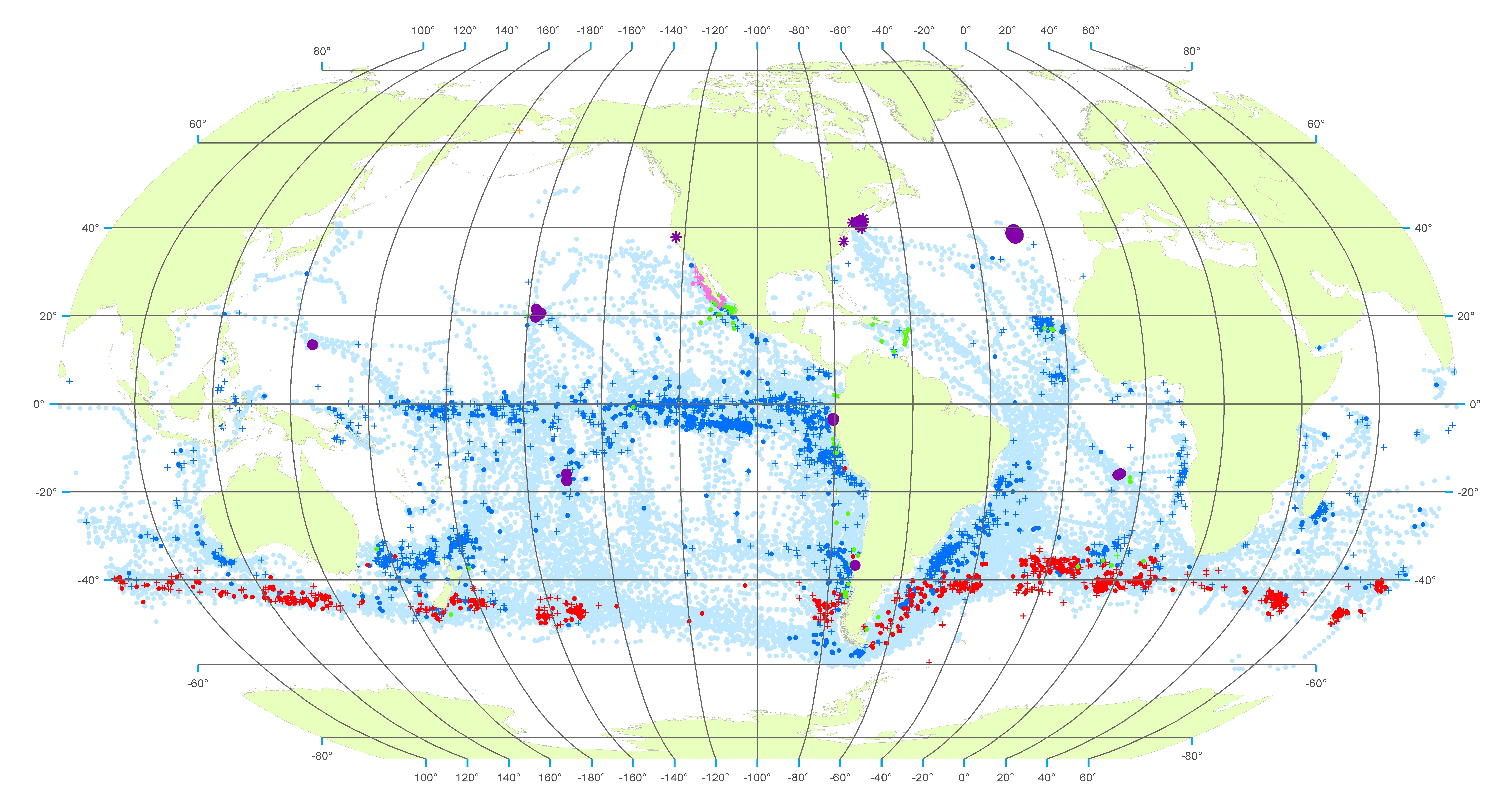

Supplement: Figure S3 — High resolution map of January observations of whales. The data were extracted from a sample of American whaling logbooks for voyages departing between 1780 and 1920. Days with no whale observations and days with observations of sperm, right, bowhead, humpback, and gray whales and locations of key ports were distinguished by the colors indicated. (JPG) [file pone.0034905.s003.jpg]

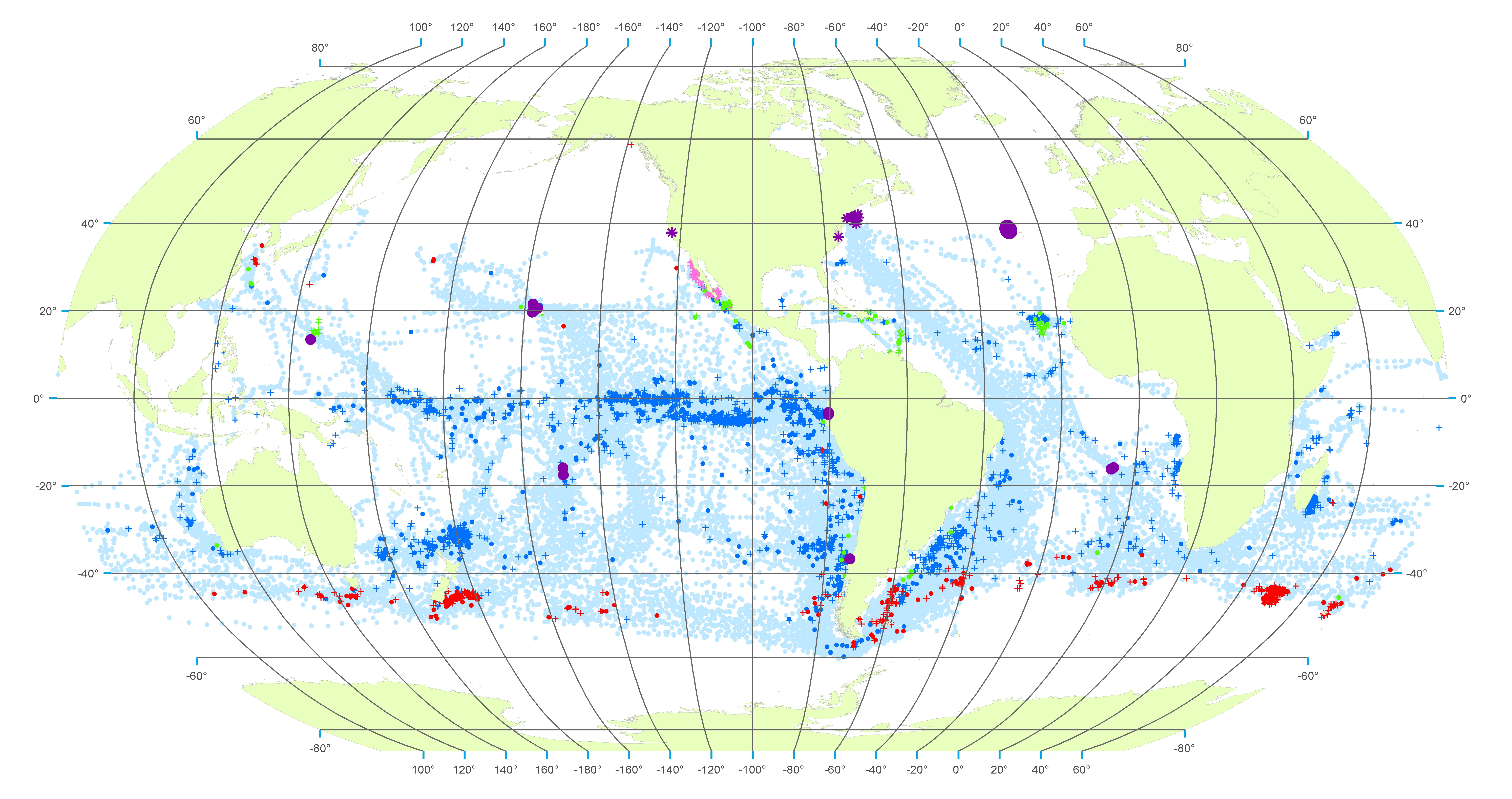

Supplement: Figure S4 — High resolution map of February observations of whales. The data were extracted from a sample of American whaling logbooks for voyages departing between 1780 and 1920. Days with no whale observations and days with observations of sperm, right, bowhead, humpback, and gray whales and locations of key ports were distinguished by the colors indicated. (JPG) [file pone.0034905.s004.jpg]

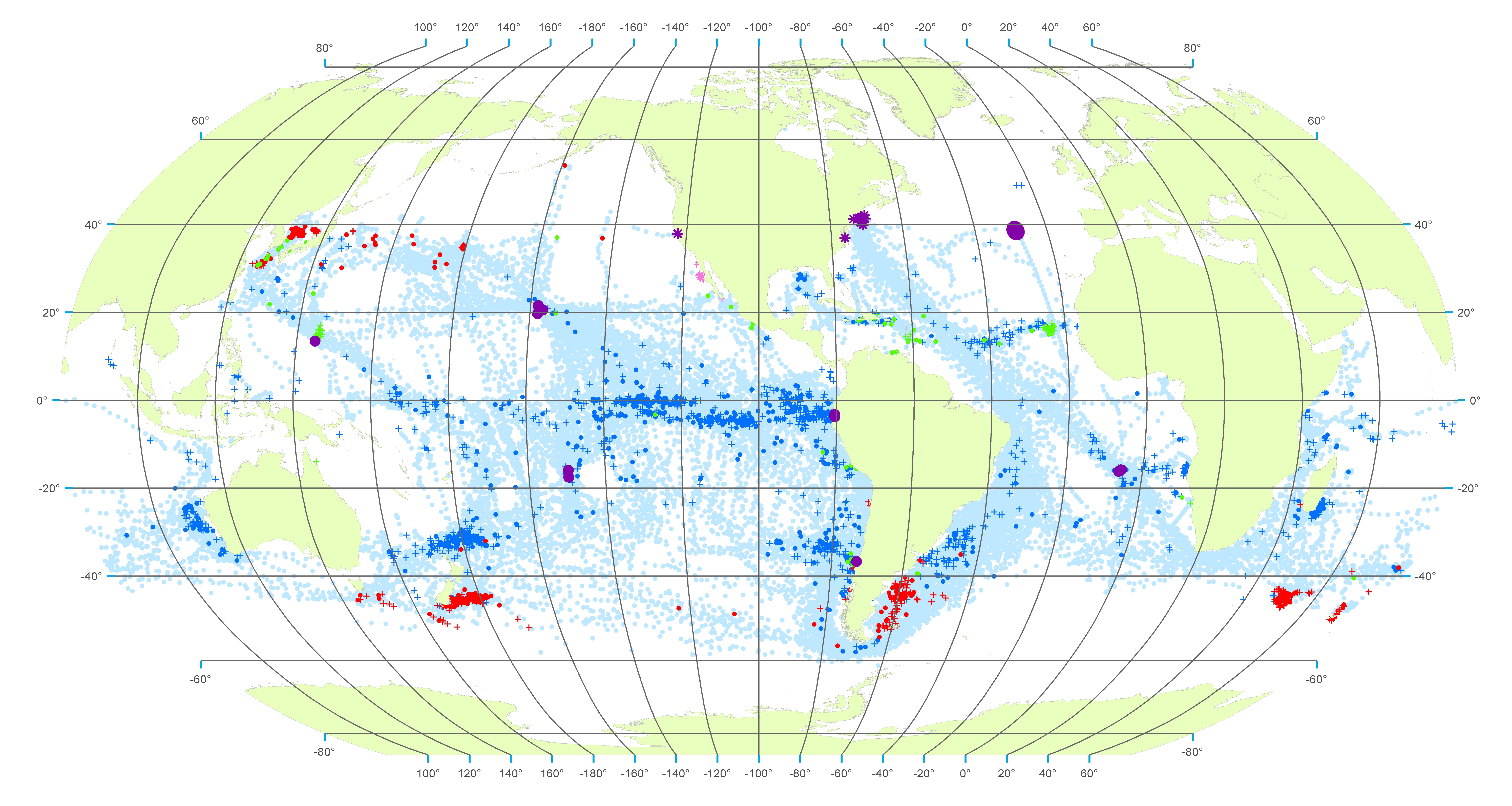

Supplement: Figure S5 — High resolution map of March observations of whales. The data were extracted from a sample of American whaling logbooks for voyages departing between 1780 and 1920. Days with no whale observations and days with observations of sperm, right, bowhead, humpback, and gray whales and locations of key ports were distinguished by the colors indicated. (JPG) [file pone.0034905.s005.jpg]

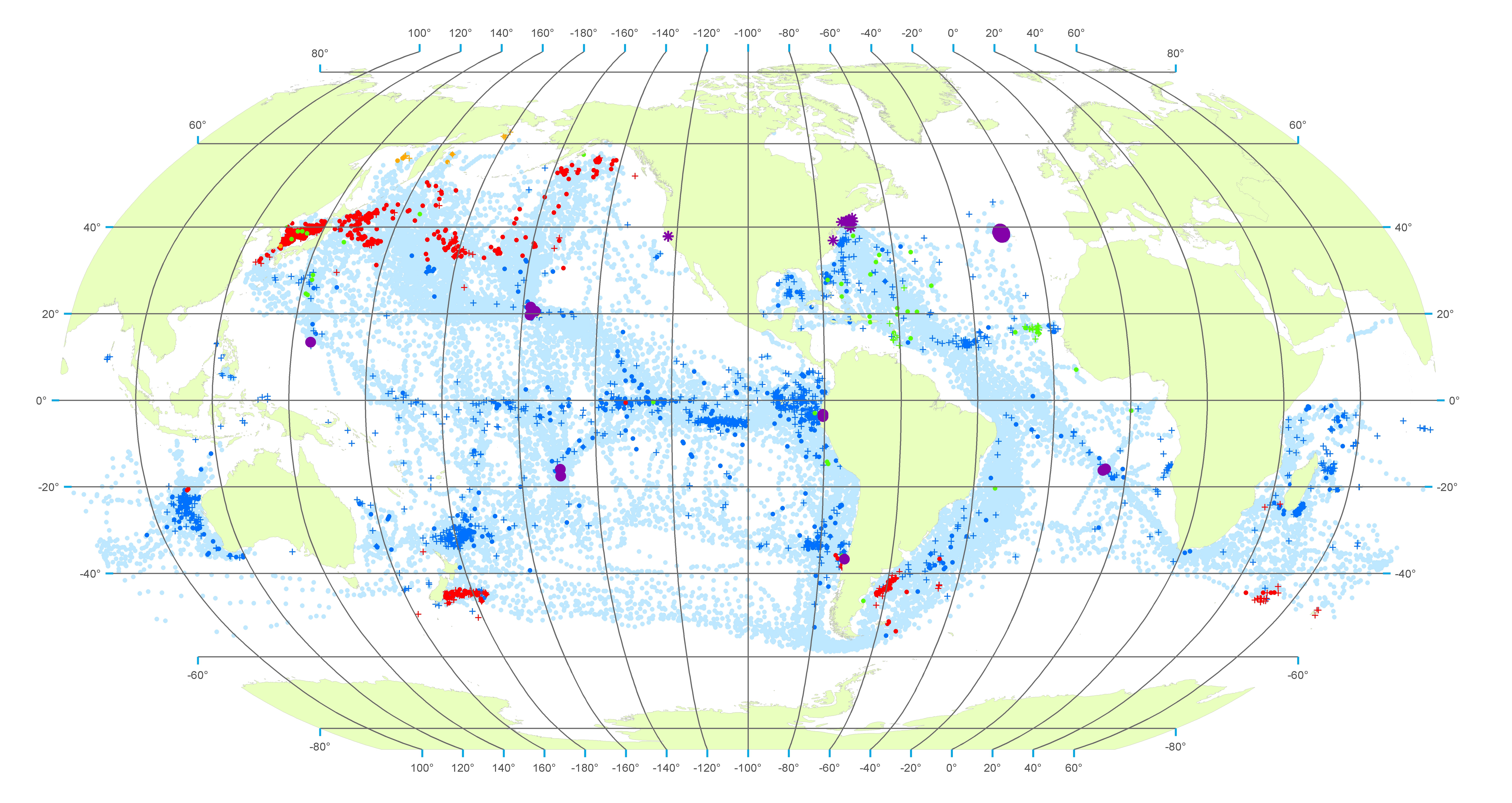

Supplement: Figure S6 — High resolution map of April observations of whales. The data were extracted from a sample of American whaling logbooks for voyages departing between 1780 and 1920. Days with no whale observations and days with observations of sperm, right, bowhead, humpback, and gray whales and locations of key ports were distinguished by the colors indicated. (JPG) [file pone.0034905.s006.jpg]

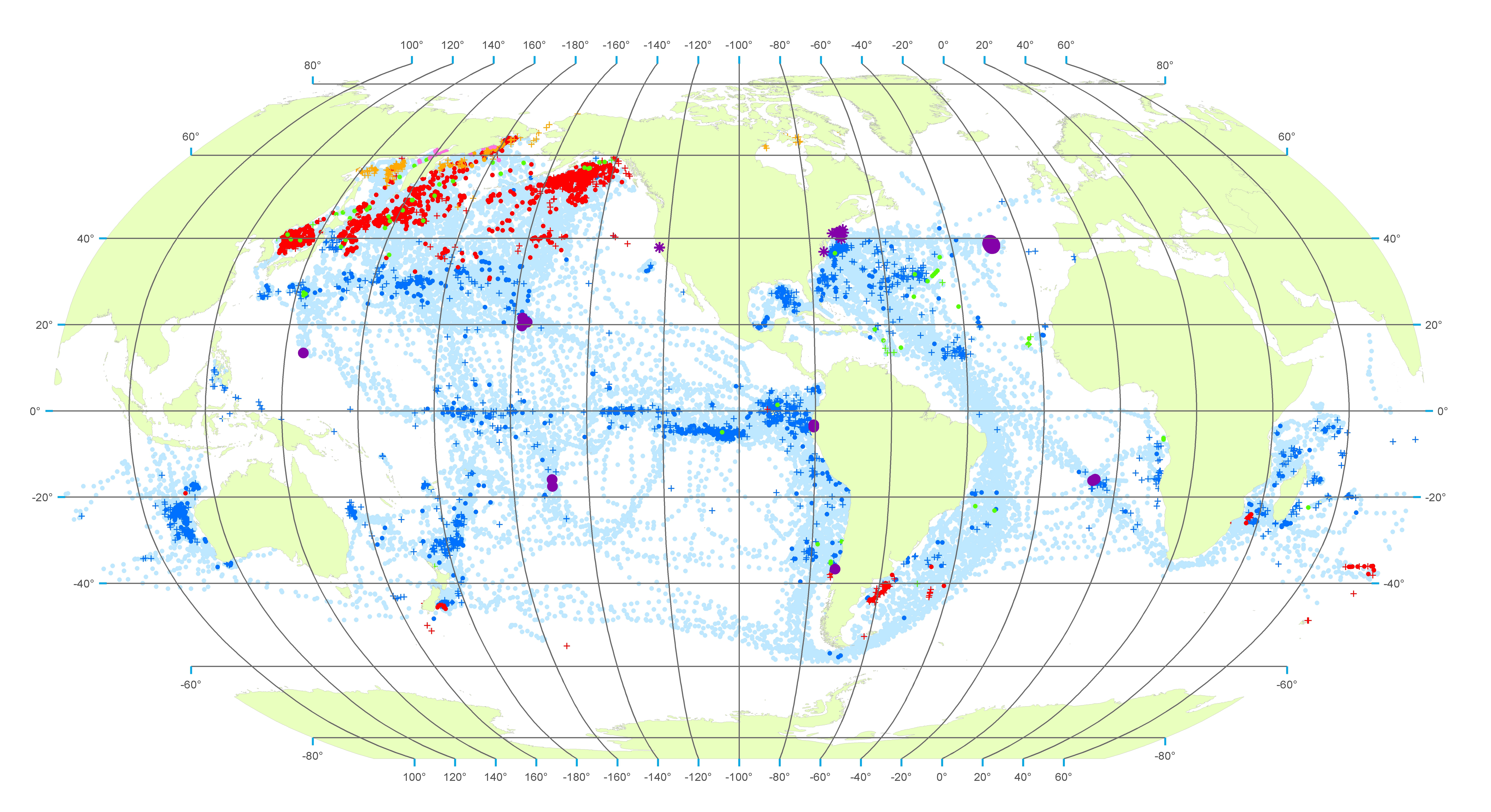

Supplement: Figure S7 — High resolution map of May observations of whales. The data were extracted from a sample of American whaling logbooks for voyages departing between 1780 and 1920. Days with no whale observations and days with observations of sperm, right, bowhead, humpback, and gray whales and locations of key ports were distinguished by the colors indicated. (JPG) [file pone.0034905.s007.jpg]

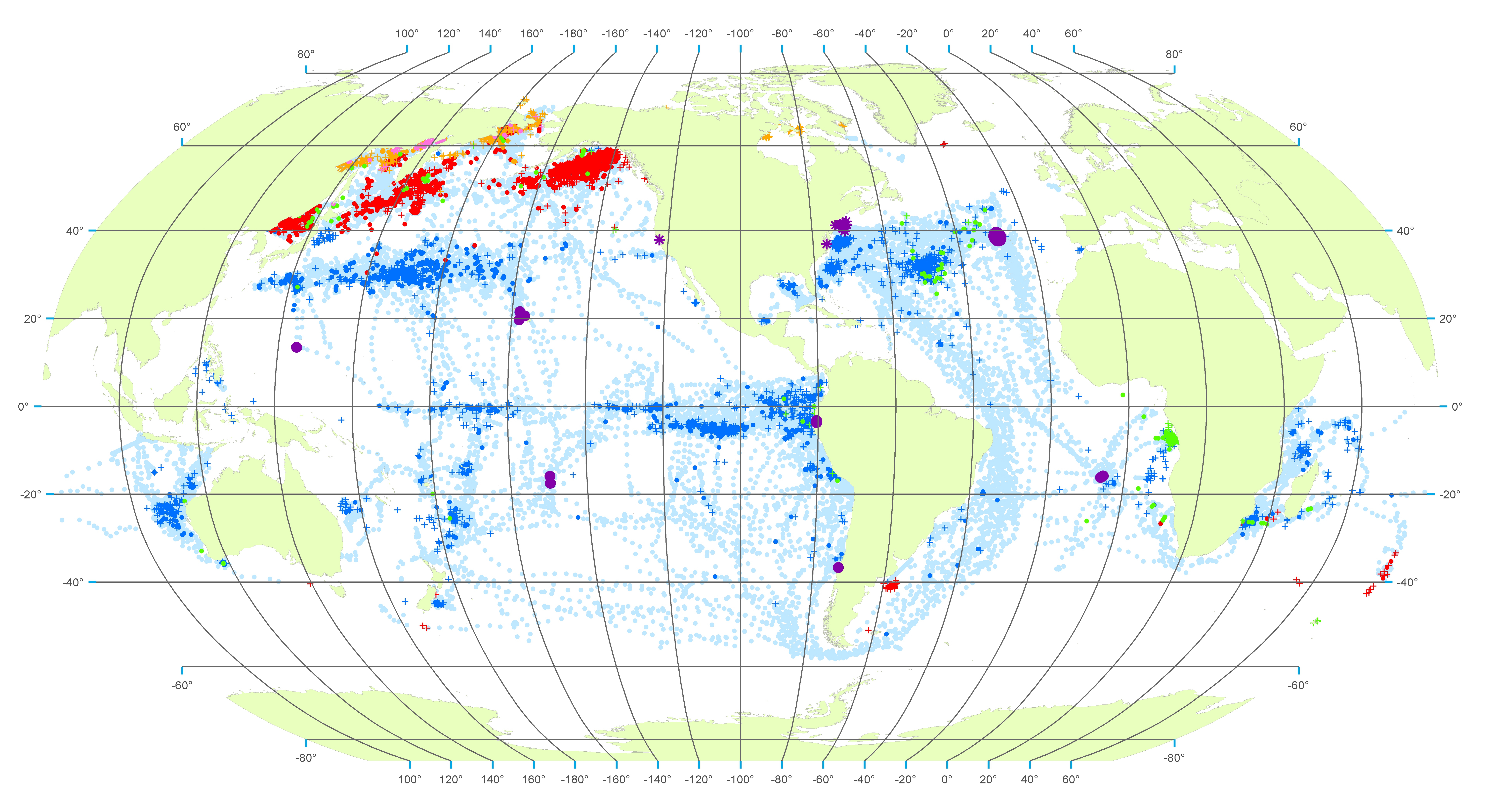

Supplement: Figure S8 — High resolution map of June observations of whales. The data were extracted from a sample of American whaling logbooks for voyages departing between 1780 and 1920. Days with no whale observations and days with observations of sperm, right, bowhead, humpback, and gray whales and locations of key ports were distinguished by the colors indicated. (JPG) [file pone.0034905.s008.jpg]

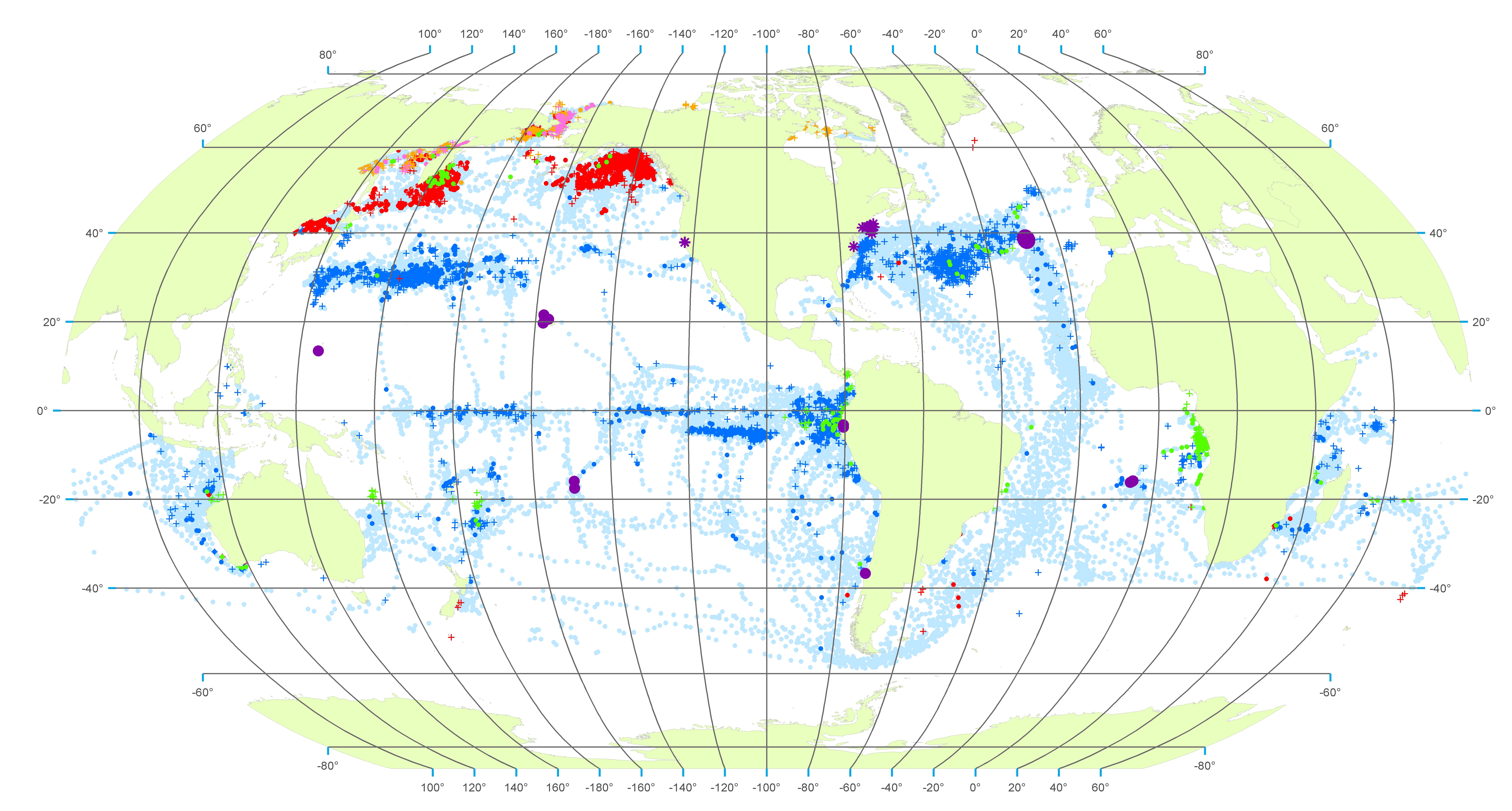

Supplement: Figure S9 — High resolution map of July observations of whales. The data were extracted from a sample of American whaling logbooks for voyages departing between 1780 and 1920. Days with no whale observations and days with observations of sperm, right, bowhead, humpback, and gray whales and locations of key ports were distinguished by the colors indicated. (JPG) [file pone.0034905.s009.jpg]

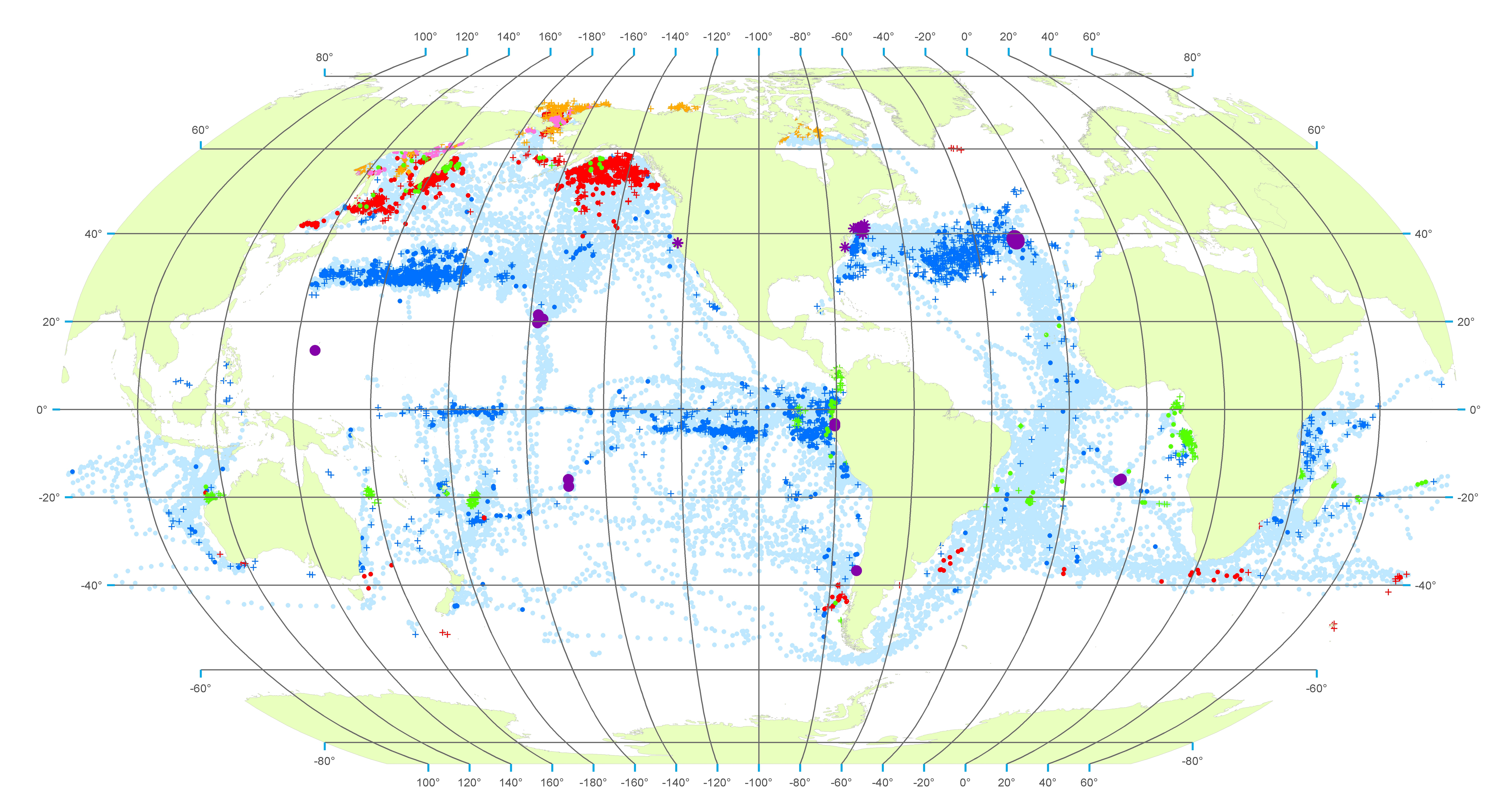

Supplement: Figure S10 — High resolution map of August observations of whales. The data were extracted from a sample of American whaling logbooks for voyages departing between 1780 and 1920. Days with no whale observations and days with observations of sperm, right, bowhead, humpback, and gray whales and locations of key ports were distinguished by the colors indicated. (JPG) [file pone.0034905.s010.jpg]

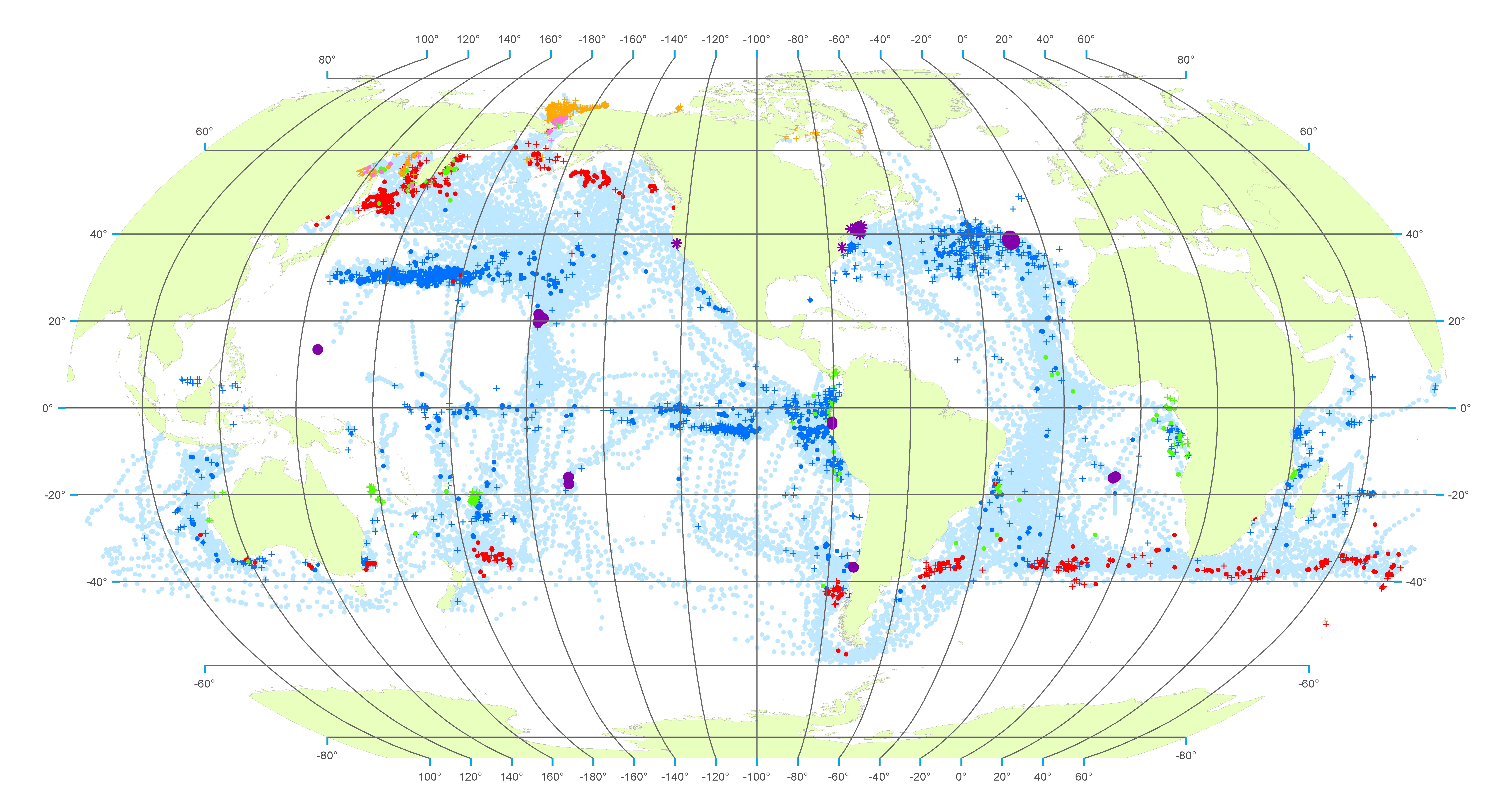

Supplement: Figure S11 — High resolution map of September observations of whales. The data were extracted from a sample of American whaling logbooks for voyages departing between 1780 and 1920. Days with no whale observations and days with observations of sperm, right, bowhead, humpback, and gray whales and locations of key ports were distinguished by the colors indicated. (JPG) [file pone.0034905.s011.jpg]

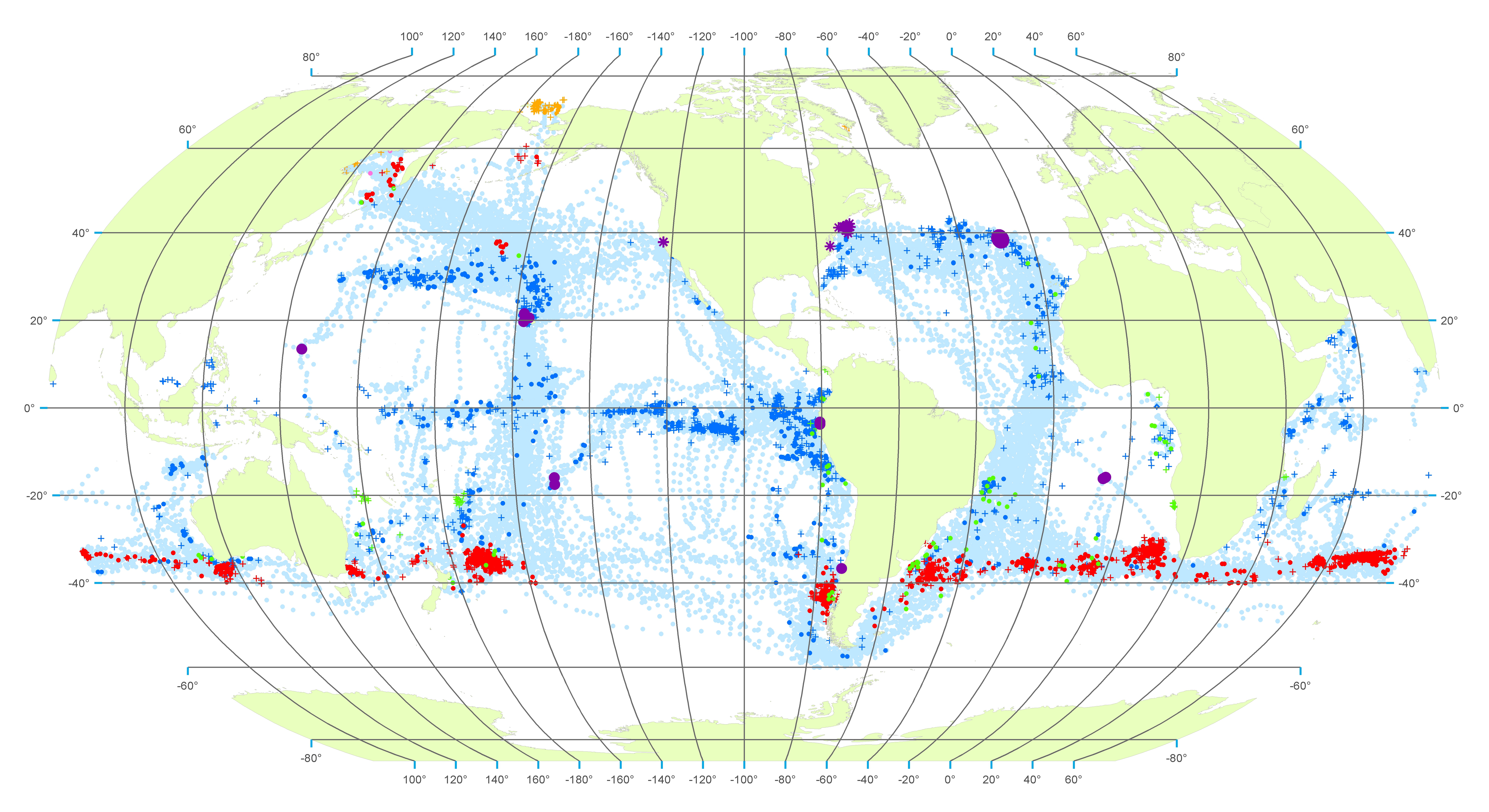

Supplement: Figure S12 — High resolution map of October observations of whales. The data were extracted from a sample of American whaling logbooks for voyages departing between 1780 and 1920. Days with no whale observations and days with observations of sperm, right, bowhead, humpback, and gray whales and locations of key ports were distinguished by the colors indicated. (JPG) [file pone.0034905.s012.jpg]

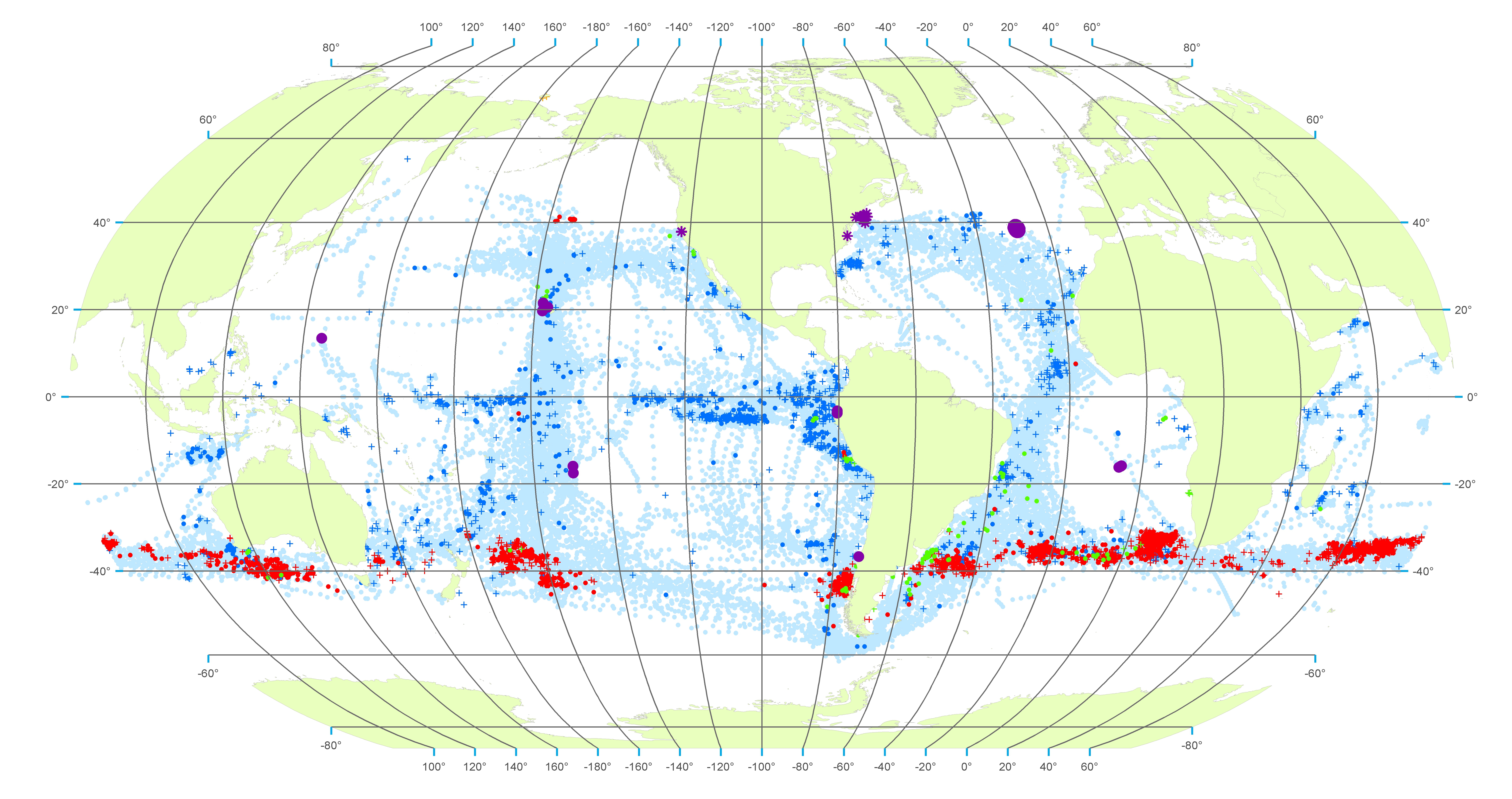

Supplement: Figure S13 — High resolution map of November observations of whales. The data were extracted from a sample of American whaling logbooks for voyages departing between 1780 and 1920. Days with no whale observations and days with observations of sperm, right, bowhead, humpback, and gray whales and locations of key ports were distinguished by the colors indicated. (JPG) [file pone.0034905.s013.jpg]
